# Transcriptome analysis of human ageing in male skin shows mid-life period of variability and central role of NF-κB

**DOI:** 10.1038/srep26846

**Published:** 2016-05-27

**Authors:** Daniel J. Haustead, Andrew Stevenson, Vishal Saxena, Fiona Marriage, Martin Firth, Robyn Silla, Lisa Martin, Katharine F. Adcroft, Suzanne Rea, Philip J. Day, Phillip Melton, Fiona M. Wood, Mark W. Fear

**Affiliations:** 1 The Fiona Wood Foundation, Perth, WA 6000, Australia; 2 Faculty of Medicine, Dentistry and Health Sciences, University of Western Australia, Crawley WA 6009, Australia; 3 Faculty of Medicine and Health Sciences, University of Manchester, Manchester, M1 7DN, UK; 4 Burn Injury Research Unit, School of Surgery, University of Western Australia, Crawley WA 6009, Australia; 5 Department of Mechanical Engineering, Massachusetts Institute of Technology, Cambridge, Massachusetts 02139, USA; 6 Burns Service of Western Australia, Royal Perth Hospital and Princess Margaret Hospital, WA 6000, Australia; 7 Centre for Genetic Origins of Health and Disease, University of Western Australia, Crawley WA , Australia 6009

## Abstract

Age is well-known to be a significant factor in both disease pathology and response to treatment, yet the molecular changes that occur with age in humans remain ill-defined. Here, using transcriptome profiling of healthy human male skin, we demonstrate that there is a period of significantly elevated, transcriptome-wide expression changes occurring predominantly in middle age. Both pre and post this period, the transcriptome appears to undergo much smaller, linear changes with increasing age. Functional analysis of the transient changes in middle age suggest a period of heightened metabolic activity and cellular damage associated with NF-kappa-B and TNF signaling pathways. Through meta-analysis we also show the presence of global, tissue independent linear transcriptome changes with age which appear to be regulated by NF-kappa-B. These results suggest that aging in human skin is associated with a critical mid-life period with widespread transcriptome changes, both preceded and proceeded by a relatively steady rate of linear change in the transcriptome. The data provides insight into molecular changes associated with normal aging and will help to better understand the increasingly important pathological changes associated with aging.

Few organs are as consciously linked to aging as skin, which through loss of elasticity and formation of wrinkles serves as a stark visual reminder of the process. However, age related changes in the skin are more than just cosmetic, with decline in function manifested by inefficiencies in physiological processes including wound healing and thermoregulation^1^. Further the extent of functional decline in internal tissues can be estimated through sampling of the skin^2^. Despite this, much remains unknown regarding the changes in the transcriptome with aging in the skin, and whether the changes observed in other organs are indicative of a universal or tissue specific aging process.

Numerous evolutionary theories of aging have attempted to explain how a universal aging process would occur. Two such theories are those of antagonistic pleiotropy and the disposable soma, which suggest an inverse relationship between reproduction and aging^3^. These theories hypothesize that aging occurs as a result of a “trade-off”. Consistent with a link between reproduction and aging are observations in model organisms that a lower rate of reproduction slows down aging. Genetically manipulated long-lived model organisms display decreased fecundity, while lifespan also increases with ablation of their germ-line or removal of the entire reproductive system^4^,^5^. Despite this, there is little evidence of a relationship between reproduction and aging in humans. Indeed, studies of human aging have largely ignored the possibility that discrete time periods, such as the reproductive period, may modulate the aging process.

These studies have instead focused on identifying progressive cumulative changes associated with damage-based theories of aging, primarily by comparing “young” and “old” individuals. The most prominent of the damage-based theories is the free radical theory of aging^6^. This theory is supported by evidence that oxidative damage accumulates with age and plays a key role in the development of aging related diseases such as atherosclerosis, malignancy and neurodegenerative diseases^7^. Similar to this is the theory that chronic low-grade inflammation results in increasing levels of tissue damage and thereby causes aging^8^. Interestingly, caloric restriction experiments support a possible link between the reproductive and damage-based theories of aging, whereby increases in lifespan are coupled with decreased fecundity, delayed progression to sexual maturation and also decreased levels of oxidative-stress and inflammation^5^,^9^,^10^.

Current understanding of the changes in gene expression associated with human aging is limited by the lack of normal healthy tissue that can be assayed. Consequently, many previous studies investigating age related changes in the transcriptome have either been conducted on samples taken from individuals with an underlying pathology, during surgery or post-mortem. This difficulty in obtaining healthy tissue has also meant that variables known to affect the rate of aging such as gender, ethnicity, smoking and medical history have largely been ignored in favor of sample size.

The external location of the skin makes it unique in that samples can be obtained from healthy volunteers without preceding activation of inflammatory responses (as is the case with, for example, surgery or post-mortem sampling). As such, this current study seeks to address the gaps in our understanding of changes in the transcriptome across the healthy adult lifespan by assessing changes in gene expression from non sun-exposed skin from healthy male volunteers aged 19–86 (Supplementary Table 1). Further, we conduct a meta-analysis of our data with previous microarray experiments using human brain, muscle and kidney to investigate the presence of a universal aging process in human males.

## Results

### Less than 1% of the skin transcriptome changes in expression between 19–29 and 70+ age groups

**Figure 1:**
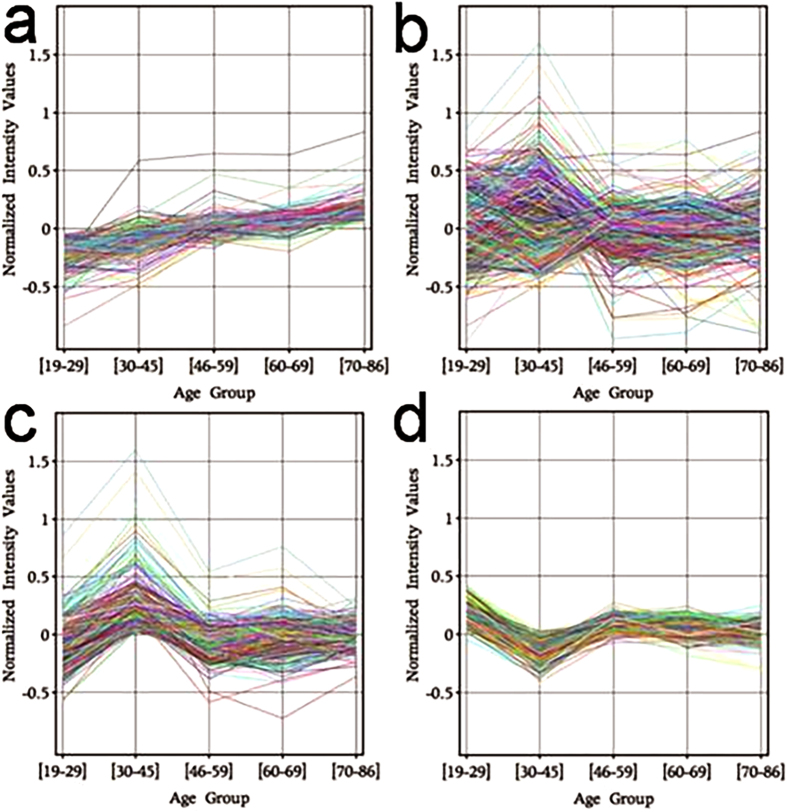
Expression of the transcriptome of human skin across the lifespan. (**a)** 174 genes undergo a gradual, progressive increase in expression with age. (**b**) The entire transcriptional profile of normal human skin from 98 individuals relative to age groups. (**c**) 2969 genes demonstrate a statistically significant transient peak in expression at age 30–45. (**d**) 556 genes show an opposing transient decrease in expression at age 30–45.

There was little change in the expression of the transcriptome between the youngest and eldest age groups, with less than 1% of the transcriptome significantly changing in expression levels when assessed using both p < 0.01 with limma analysis and false discovery rate (FDR) <5% with significance analysis of microarray (SAM) analysis. Of the 174 genes that had significantly altered expression levels between the youngest (22.72 ± 2.14) and eldest (74.58 ± 3.7) age groups, all demonstrated a small (max 2.9-fold, mean 1.5-fold) increase in expression (Supplementary Data, Table 2). Further analysis revealed that the increase in expression occurred in a predominantly linear pattern with increasing age (Fig. 1a).

Surprisingly, there was no significant decrease in the expression of any genes between the youngest and eldest age groups when using the original parameters described above. Indeed, even when removing the limitation that the gene must be identified by both SAM and limma analysis, only six genes were identified that demonstrated a significant age-related decline in expression.

### Increasing age correlates with an increase in expression of cellular stress response pathways

Identification of multiple genes with related functions suggests greater confidence in pathway changes than can be placed on individual gene results^11^. Gene ontology (GO) analysis revealed an upregulation of functional categories predominately related to the immune response, yet no observed corresponding increase in expression of genes associated with cell repair (Supplementary Data Table 3). Gene set enrichment analysis (GSEA) results support this, with an age-related enrichment for multiple inflammation and stress activated pathways but no corresponding enrichment for repair mechanisms (Supplementary Data Table 3). This suggests that the observed age-related changes in physiological processes in human skin (such as wound healing) have a temporal correlation with underlying genetic changes suggestive of increasing inflammation and cellular stress.

### Gene-gene relationships implicate *TGFB1* and *TNF* in the aging process

**Figure 2:**
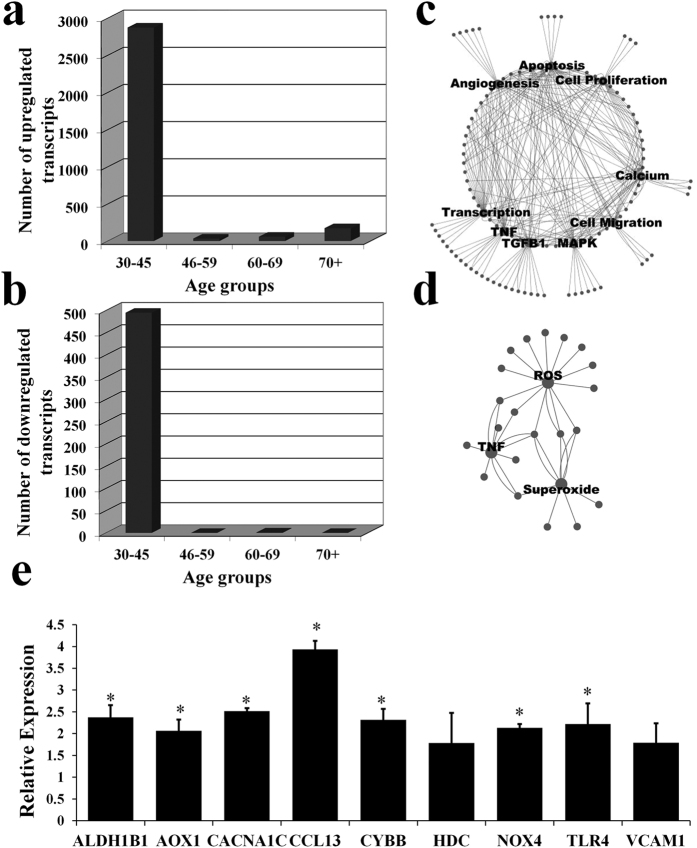
(**a**) Number of genes significantly upregulated in each age group. (**b**) Number of genes significantly downregulated in each age group. (**c**) Interaction network of common factors regulating or regulated by genes identified as upregulated at age 70+. (**d**) Interaction network of common factors involved in the metabolism of those genes identified as upregulated at age 70+. (**e**) qPCR results for a subset of genes identified as upregulated with age and implicated in the stress and/or apoptotic response. Results indicate relative expression in age 70+ compared with age 19–29. Error bars represent standard error of the mean. (*P ≤ 0.05).

Interaction networks were constructed and indicate that transforming growth factor beta 1 (*TGFB1)*, tumor necrosis factor (*TNF)* and mitogen activated protein kinases (MAPK) are known to be regulated by and central to the regulation of the expression of the majority of the genes identified as increasing in expression with age (Fig. 2c). Pathways built using Pathway studiomammal^®^(Elsevier) to identify common regulators also identified *TGFB1* and *TNF* as genes central to the changes observed when comparing the eldest age group to the youngest age group (Fig. S1a). As such, the age-related increase in expression of many of the genes identified could predominantly be explained by changes in the levels of *TGFB1* and *TNF*.

Another approach to identifying genes of importance is to determine physiological processes regulated by the identified genes and then ascertain if any of the genes are common to these processes. The constructed interaction network implicates age-regulated genes as central to the regulation of apoptosis, angiogenesis, cell proliferation and migration (Fig. 2c). 18 genes provide a common link to these processes and as such may be of particular interest for future studies in model organisms. Specifically; calcium channel, voltage-dependent, L type, alpha 1C subunit (*CACNA1C)*, chemokine (C-C motif) receptor 1 (*CCR1)*, complement factor H (*CFH)*, cysteine and glycine-rich protein 1 (*CSRP1)*, connective tissue growth factor (*CTGF)*, cytochrome b-245, beta polypeptide (*CYBB)*, ETS-domain protein (*ELK3)*, filamin A, alpha (*FLNA)*, NADPH oxidase 4 (*NOX4)*, neuropilin 1 (*NRP1)*, tissue factor pathway inhibitor (*TFPI)*, transforming growth factor, beta receptor 1 *(TGFβR1)*, thrombospondin 1 (*THBS1)*, thrombospondin 2 (*THBS2)*, TIMP metallopeptidase inhibitor 3 (*TIMP3)*, toll-like receptor 4 (*TLR4)*, vascular cell adhesion molecule 1 (*VCAM1)*, versican (*VCAN)*.

An interaction network of age-regulated genes and their associated metabolites shows two small clusters centred on the production and inhibition of superoxide and reactive oxygen species (ROS) (Fig. 2d). This suggests that not only do the identified genes respond to increasing levels of cellular stress, but can also contribute to it.

### qPCR confirms age-related changes identified in microarray expression data

To validate our findings, we used quantitative real-time PCR (qPCR) for a subset of genes present in the above list of “core” genes of interest and/or displaying a relationship with ROS metabolism. A minimum of 5 individual samples from each age group were used to test expression levels of each gene. Results from qPCR revealed a significant (*P* ≤ 0.05) increase in expression with age in seven of the nine genes tested, with the remaining two genes demonstrating a smaller increase which did not reach statistical significance (Fig. 2e).

### The 30–45 age group is associated with a transient period of transcriptome variability in skin

Comparison of the transcriptome in each age group showed that between the ages of 30–45 (39.67 ± 4.85) there is a substantial change not seen in any other age group, with expression levels of over 25% of the transcriptome changing relative to individuals from all other age groups (Fig. 1b). These changes predominately represented an increase in expression (2872 genes), with a smaller number of genes undergoing a decrease in expression (495 genes) (Fig. 2a,b). The significant difference between this group and all other groups is highlighted using heatmaps to show gene expression levels of those genes both up (Fig. S2a) and down (Fig. S2b) regulated in the 30–45 age group across the five age groups that were compared. Mapping of the 2872 genes upregulated at age 30–45 (Fig. 1c) and the 495 genes downregulated at age 30–45 (Fig. 1d) revealed the period of transcriptional change at age 30–45 to be transient in nature. Indeed, over 95% of the genes identified as differentially expressed at age 30–45, returned by age 46–59 to the same level as those at age 19–29.

Clustering of each individual expression dataset using the genes differentially expressed in the 30–45 age group shows a core of 8 individuals from this group of 18 closely aligned (Fig. S3a,b). Samples from other age groups are also similar to this core group. However only 1 sample from the youngest and 1 sample from the eldest age group is closely aligned with this group, suggesting the changes, whilst likely occurring over a wider age group than the 30–45 years grouped here, are transient and associated with the mid-life period of ageing.

### The change in the transcriptome at age 30–45 is consistent with a period of heightened metabolic activity and cellular damage

GO and GSEA analysis of those genes upregulated in the 30–45 age group revealed an over-representation of genes involved in various aspects of biopolymer, protein and cell metabolism, the cell cycle and post-translational protein modification, consistent with a transient increase in metabolic activity (Supplementary data Table 4a,c). The upregulation of genes involved in RNA degradation, response to DNA damage stimulus and the positive regulation of the I-κB kinase/NF-κB cascade suggests the presence of significant cellular modification and genetic damage during this period of increased metabolic activity. Contrary to what would be expected, there was a significant negative enrichment for compensatory mechanisms such as telomere maintenance, numerous processes related to growth and development, oxidoreductase activity and beta-oxidation (Supplementary Data Table 4b,c).

### *TNF* and *TP53* are key common regulators of expression changes observed in the mid-life period

Constructed interaction networks reveal that tumor protein p53 (*TP53)* and *TNF* have known roles in the regulation of expression, binding and protein modification of a large number of the genes spiking in expression at age 30–45 (Fig. 2a,b). Pathways used to identify common regulators also show *TNF* and *TP53* are critical regulators of many of the genes changing in expression during this period (Fig. S1b). Similar to the changes seen in the oldest age group, *TGFβ* is also a common regulator. Interestingly, the insulin gene (*INS*) also appears to be a common regulator of many of the genes altered in expression during mid-life. Both *INS* and *TP53* are increased in expression during this time. In stark contrast to the comparison of the eldest age group changes, both *TGFβ* and *TNF* are decreased in expression and this is reflected by large numbers of connected genes also decreasing in expression.

**Figure 3:**
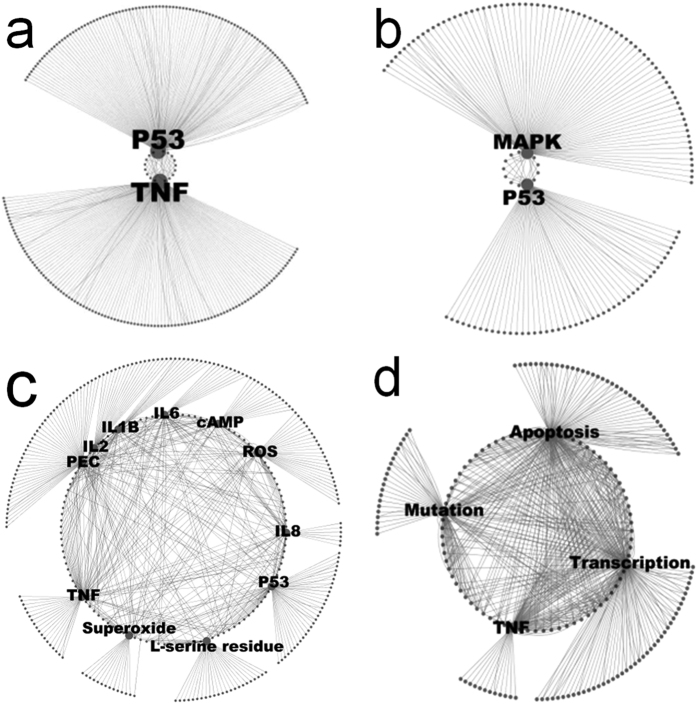
(**a)** Interaction network of common factors involved in the expression of genes peaking in expression at age 30–45. (**b**) Interaction network of common factors in the protein modification of genes peaking in expression at age 30–45. (**c**) Metabolite profile of genes peaking in expression at age 30–45. (**d**) Interaction network of common factors regulating or regulated by genes identified as downregulated at age 30–45.

The metabolic profile of the genes upregulated in the mid-life period suggests the cellular effects of this heightened period of metabolic activity are likely mediated through increased production of superoxide, ROS and interleukins 1B, 2, 6 and 8 (Fig. 3c). Despite this, an interaction network from genes identified as downregulated in this time period indicates a TNF regulated decrease in compensatory mechanisms such as apoptosis and transcription (Fig. 3d). Therefore a consistent pattern emerges through this data suggestive of increased genetic damage coupled with decreased compensatory mechanisms in this mid-life period.

### qPCR confirms identified microarray expression changes in the mid-life period

**Figure 4:**
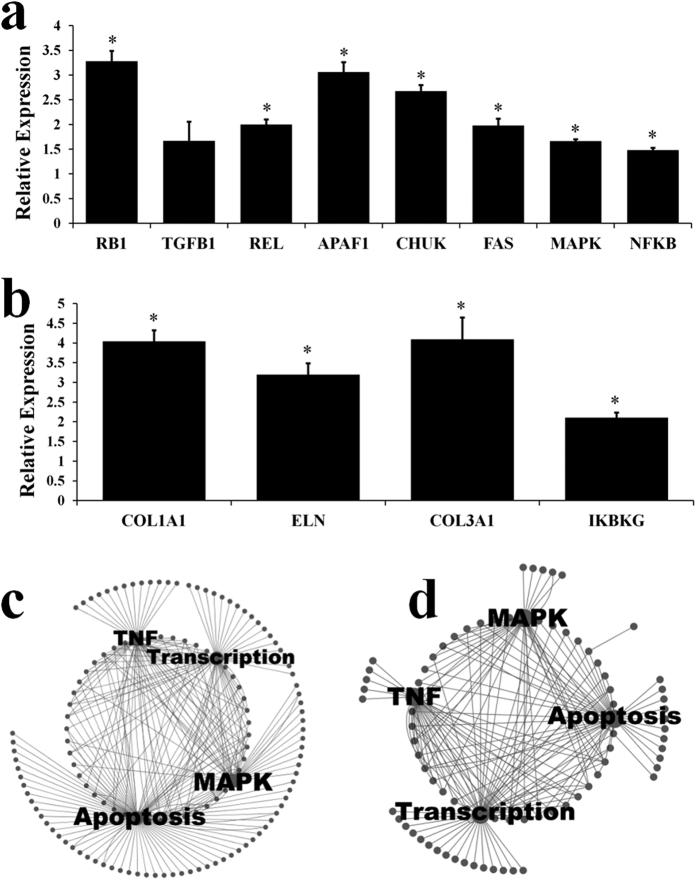
(**a**) qPCR results for a subset of genes identified as transiently upregulated at age 30–45. Results indicate relative expression at age 30–45 compared with age 19–29. Error bars represent standard error of the mean. (*P ≤ 0.05) (**b**) qPCR results for a subset of genes identified as transiently downregulated at age 30–45. Results indicate relative expression at age 19–29 compared with age 30–45. Error bars represent standard error of the mean. (*P ≤ 0.05) (**c**) Interaction network of common factors regulating or regulated by genes identified as upregulated with a regression meta-analysis across the lifespan in male data. (**d**) Interaction network of common factors regulating or regulated by genes identified as upregulated at age 70+ in a meta-analysis of males aged 70+ compared with age 18–29.

Again qPCR was used to validate our microarray findings, this time for a selection of genes playing a central role in the period of mid-life variability or of structural importance in skin and observed to be significantly elevated or decreased in the microarray experiment. As for the previous analysis, a minimum of 5 individuals from each age group (youngest and 30–45) were used and tested individually for each gene (no samples were pooled). Seven of the eight genes identified as upregulated in the 30–45 year old age group in the microarray analysis and subsequently tested with qPCR demonstrated a significant (*P* ≤ 0.05) increase in expression at age 30–45 with qPCR (Fig. 4a). Four genes identified as transiently decreasing in expression at age 30–45 were tested and all showed a significant (*P* ≤ 0.05) decrease in expression at age 30–45 (Fig. 4b). This supports the validity of the microarray data.

### A similar period of transcriptome variability is observed in human entorhinal cortex

To determine whether the period of transcriptome variability we observed at age 30–45 was specific to skin, we re-assessed previous human aging microarray studies from four brain regions^12^, the kidney cortex and medulla^13^ and two skeletal muscle sites^14^,^15^. A similar period of transcriptional variability at age 30–45 was observed in the human entorhinal cortex with 960 genes differentially expressed when compared to all other age groups at age 30–45, compared with 138 at age 46–59 and 173 at age 70+. Other regions of the brain assessed (posterior central gyrus, superior frontal gyrus and hippocampus) all showed increasing numbers of differentially expressed genes with increasing age, as did skeletal muscle. In contrast, the kidney analysis demonstrated few unique transcriptional changes in each age group.

### Meta-analysis of aging human microarray data sets identifies underlying transcriptome changes independent of tissue type

Using GSEA we compared gene sets taken from the results of previous human aging microarray studies^12^,^13^,^14^,^15^,^16^ with our microarray data. Genes identified as upregulated with age in previous studies demonstrated a strong positive correlation with increasing age in our data, regardless of the ages of the youngest individuals, gender, or tissue assayed (Q < 0.05) (Table 1). This suggests the presence of a common underlying aging process present in human skin, brain, kidney and muscle. In contrast, none of the previously identified downregulated gene sets demonstrated a significant age-related negative enrichment in our data. Indeed, those gene sets constructed from genes identified in studies where the youngest individuals were predominantly aged 30–45, showed an unexpected weak upregulation with increasing age. Expression of individual genes identified as downregulated with age in previous studies were mapped across age-groups in our skin expression data. This revealed that genes identified as downregulated in studies defining the youngest individuals as aged 19–29 were more likely to decrease in expression between ages 19–29 and 70+ in skin (P = 0.004), and more likely to display a progressive decline in expression across the entire lifespan in skin (P = 0.00009). In contrast, genes identified as downregulated in studies defining the youngest individuals as aged 30–45 were better able to identify genes decreasing in expression between ages 30–45 and 70+ in skin (P = 0.0007). However, 96.3–99.3% of the genes identified as downregulated with age in studies defining the youngest individuals as aged 30–45 demonstrated a transient peak in expression at age 30–45 in skin, significantly more than in studies defining the youngest individuals as aged 19–29 (P = 0.0003).

### The majority of the transcriptome remains unchanged with increasing age

Similar to our observations in skin, the expression of the transcriptome across multiple tissues appears predominantly stable with age, with only a small percentage of the transcriptome changing expression with advancing age. Specifically, the regression analysis identified changes in approximately 1% of the transcriptome (248 genes upregulated with increasing age and 191 downregulated) and even less in the direct comparison of 19–29 year olds with 70+ year olds, with 129 genes showing increased expression in the 70+ age group and 154 with a decreased expression (Fig. 4a).

### There is a progressive increase in immune system processes and the I-κB kinase/NF-κB cascade with increasing age

GO and GSEA were applied to functionally characterize the genes identified as changing in expression with age. This revealed a consistent upregulation of the immune system process above all other functional categories (Supplementary Data Table 5a,b). This upregulation encompassed all aspects of the immune system process including the response to wounding/stress, activation of the immune response and antigen processing and presentation. Regression analysis confirmed that the change in the expression of the immune system process occurred continuously throughout the human lifespan.

Further, constructed interaction networks suggested a key role for both MAPK and the known NF-kappa-B activator TNF in the regulation of the genes identified as upregulated with age (Fig. 3c,d).

### Negative regulation of apoptosis, transcription, cell proliferation and metabolism increases with aging

Functional analysis, supported by constructed interaction networks also revealed an increase in expression of genes involved in oxidoreductase activity and the negative regulation of; apoptosis, transcription, cell proliferation and numerous metabolic processes. These processes undergo a progressive change in expression throughout the human lifespan with changes already apparent in early adult life (Supplementary Table 5a,b).

## Discussion

In this study we found that the transcriptome of the skin is relatively stable with age, with less than one percent of all genes changing significantly in expression level with increasing age. Of those genes that changed in expression with age, almost all demonstrated a small, approximately linear increase in expression with age, suggestive of a progressive, cumulative change in the transcriptome which would support damage-based theories of aging^17^. Even using less stringent cut-offs, only 6 genes revealed a significant decrease in expression with age, two of which (*COL1A1* and *COL3A1*) have previously been shown to decrease in expression with age in a qPCR validated expression assay in mouse lung tissue^18^. Although previous microarray studies of human aging have also identified only a small percentage of genes undergoing a significant change in expression with age, our analysis revealed a smaller number of genes changing in expression, and at a smaller magnitude. Further, the absence or very limited number of genes that decrease in expression with age in male skin is in contrast to observations in other tissues. This suggests that either the transcriptome of male skin is less affected by advancing age than internal organs, or may be a reflection of improved sample homogeneity, due to the healthy status of tissue donors. Alternatively, as ours and most other comparative microarray studies of human aging have used whole tissue biopsies rather than cell culture, it is possible that internal organs undergo a greater change in cell populations with age than male skin, which may account for the relative apparent stability of the transcriptome in male skin.

It is assumed in this study that the cell composition of the skin will not change significantly with age. Through the use of these mixed cell biopsies, the largest cell population will be keratinocytes (epithelial cells) and it is likely this will be the dominant cell type accounting for the transcriptional profiles observed. Nevertheless, changes in immune cell infiltrate, fibroblast activity or other cell types (including endothelial and melanocyte) could all be contributing to the observed changes.

Functional analysis revealed an age-related enrichment for multiple inflammation and stress activated pathways, indicating that increasing age is associated with changes in the skin which result in a progressive increase in the activation of the immune system.

This supports the growing body of evidence that aging is associated with increased inflammation^19^. The lack of an observed corresponding increase in expression of genes associated with cell repair suggests the body is unable to adequately respond to the increasing inflammation. These changes are of particular interest in the context of age-related changes in physiological processes such as human skin wound healing, whereby advancing age is associated with both slower wound healing and an increased inflammatory response to wounding^1^. Further linking aging and wound healing was the identification via gene-gene relationships that two key members of the wound healing process, *TNF* and *TGFB1*, likely play a central role in the regulation of expression of the genes identified as changing with age.

In this study we also identified a transient period of transcriptome variability occurring predominantly in individuals in the 30–45 age group. Notably, the 30–45 age group is a period of time at which reproductive fitness begins to decline in humans. Given the observed relationship between reproduction and aging in model organisms, this raises the possibility of a similar relationship between reproduction and aging in humans^5^. Functional analysis indicates that this period is associated with an increase in metabolic activity and cellular damage and a reduction in compensatory mechanisms. This supports the theory of the importance of damage-accumulation in human aging and suggests a possible transient period of accelerated damage in early mid-life. Interestingly the pathway analysis showed that during this period expression networks that were changed largely overlapped with those that changed when comparing old and young age groups. However, the direction of this change was different in the mid-life period, with many of the genes that were observed to increase in expression in the eldest age group compared to the youngest were in fact decreased in expression in the 30–45 group. This is an important observation for studies of ageing comparing different age groups as well as suggesting this mid-life period may be important in the ageing process. The identification of the *INS* gene as being a key regulator of the changes in mid-life also supports their relevance to aging, given the key role insulin is thought to play in the aging process.

These findings are surprising and contrast with the commonly accepted view that age-related gene expression changes are progressive throughout life. Analysis of previous microarray studies of human aging revealed a similar period of transcriptional variability at age 30–45 in the entorhinal cortex of the human brain, but not in other brain regions or skeletal muscle, where changes predominately occurred in the eldest age group, or in kidney medulla or cortex where there was an even distribution in each age group (although the distribution of ages in these studies were such that analysis was not optimal for a stand-alone 30–45 year old age group). These starkly contrasting results for different tissues, and even different parts of the same tissue in the case of brain, may reflect the different roles of each cell or tissue type. Indeed the entorhinal cortex contains neurons that are particularly susceptible to aging and critical to the progression of Alzheimer’s disease, which may reflect the transient period of change observed in these neurons^20^, whilst the effects of physical exertion over time on skeletal muscle would be expected to exert a toll and potentially increase dysregulation.

Another interesting observation is that in many of these tissues there is an increase in expression of large numbers of genes and many fewer genes decrease in expression. Even in the skin, whilst limited numbers increase in expression, no observed downregulation occurs. This may reflect in part the decreased chromatin integrity and reduced capability to maintain homeostasis, as observed with epigenetic changes and widespread demethylation observed with aging^21^,^22^.

Functional and pathway analysis demonstrated that the genes changing in expression in the mid-life period are most closely linked to *TP53*, *TNF*, *TGFβ* and *INS* regulation. The identification of *INS* as a key regulator in this time period is interesting given the well described role of insulin in regulating ageing^23^,^24^,^25^. In particular the strong links between these pathways and the processes observed to be affected, including stress, apoptotic pathways and free radical production suggest that this period of life is very important to the ageing process. The potential involvement of *TP53* is also of particular interest given its role as an antagonistically pleiotropic gene and the accelerated aging demonstrated in some constitutively activated *TP53* mutant mice^26^.

Interestingly, a recent study of the transcriptome in female skin from mid-life onwards demonstrated over ten times the number of differentially expressed genes than we saw in male skin^27^. It is difficult to know whether this considerable difference in the level of change in the transcriptome with age in male and female skin is simply due to factors known to affect female skin such as exogenous hormones, the menstrual cycle and menopause^28^,^29^. Alternatively, given the youngest individuals in the study of female skin fall within the age of mid-life variability identified in our study, it is possible that this would account for the significant discrepancy. Indeed, excluding the individuals aged 19–29 from our study results in a similar number of differentially expressed genes as those identified in the study of female skin. Further, the results of the functional analysis of the differentially expressed genes with age in female skin was more in line with those of our functional analysis of genes transiently changing in expression in middle age than with the largely inflammation dominant changes seen in both male skin and previous studies of other organs from early to late adult life.

One pivotal, unanswered question in human aging is whether aging is a tissue specific process. Previous microarray studies in human muscle, brain and kidney have identified some overlap in expression changes to a small number of pathways but have not convincingly demonstrated the presence of common age related gene expression changes^12^,^13^,^14^,^15^,^16^. However, these previous studies have generally focused on the eldest individuals, with large variation in the ages of the “young” control group of individuals. As such, we hypothesised that inconsistencies in the definition of “young” individuals may account for the poor correlation of results. With regards to genes that increase in expression with age, we found this not to be the case, with a strong correlation between studies, irrespective of age. This suggests the presence of a universal, progressive aging process beginning in early adult life. However, genes identified as decreasing in expression with age in previous studies did show a difference in correlation depending upon the age of the youngest individuals. Interestingly, we found that significantly more (96.3–99.3%, P = 0.0003) of the genes identified as downregulated with age in studies where the youngest individuals were aged 30–45, demonstrated a transient peak in expression at age 30–45 in our data. While it may simply be that the downregulation of genes with age is a tissue specific phenomenon, these results also raise the possibility that identification of genes downregulated with age in these studies may actually represent sampling of genes that transiently peak in expression at 30–45 years.

Meta-analysis to identify universal gene expression changes indicate that in males the default situation is one in which gene expression remains largely stable with increasing age. However some of this apparent stability may be accounted for by the known increase in inter-individual transcriptional variability with age^30^,^31^. The most significant change observed in all tissues is the gradual increase in immune system processes across the lifespan. The upregulation of genes related to the I-κB kinase/NF-κB cascade and the identification via gene-gene relationships of a central role for *TNF* provide a potential link to the upregulation of the immune system processes given their key involvement in the regulation of these responses. Supporting an upregulation of NF-kappa-B with increasing age are observations of increased NF-kappa-B activity in the skin, liver, kidney, brain, skeletal and cardiac muscle of aged rodents^32^,^33^ and vascular endothelium of aged humans^34^. Further, in keeping with what would be expected from our results, previous studies of human fibroblasts taken from skin samples from donors across the human lifespan reveal increased NF-kappa-B binding activity from middle-age onwards^35^.

NF-kappa-B again provides a potential central regulatory link to identified changes in processes such as apoptosis and cell proliferation. Indeed, the role for NF-kappa-B in regulating apoptosis is well established^36^. The likely central regulatory role of NF-kappa-B in aging male skin is strongly supported by previous work in mammals that has shown that topical inhibition of NF-kappa-B in aged murine epidermis is able to reverse both the aging phenotype and a significant portion of the changes in the transcriptome with age, with a resultant increase in cell proliferation^37^. Further, recent studies show that systemic NF-kappa-B inhibition is able to delay the aging phenotype (including changes in skin thickness) in both progeroid and wild-type mice and reduce oxidative DNA damage and stress and delayed cellular senescence^38^,^39^. As such this data suggests that there is a universal aging process with a central regulatory role for NF-kappa-B and associated with a progressive increase in expression of genes involved in the immune system process and the negative regulation of; apoptosis, transcription, cell proliferation and metabolism

The transcriptome data suggests control of expression becomes more permissive with age, rather than a progressive increase in gene switching off. This may reflect loss of transcriptional regulation as a result of cell damage over time, and the larger number of genes that increase with expression with age, particularly in regions of the brain or skeletal muscle, strongly suggests a lack of widespread switching off of gene expression.

The results presented here provide some evidence for the mid-life period being particularly important for ageing. This adds some weight to the links suggested between reproduction and the ageing process. However, much of the data presented here shows changes in processes linked to cumulative damage over time. In addition, this study is based solely on transcriptome changes observed using microarrays and confirmed using qPCR. Additional protein based studies to demonstrate changes will be critical to confirm the findings presented here.

What is striking is the overlap between the pathways changed in both the mid-life time period and cumulatively with age. This suggests these pathways may be important at different stages of the ageing process and has implications for studies of ageing in humans as well as the possibility of future intervention.

## Methods

### Ethics approval

This study was carried out in accordance with the regulations outlined by the NHMRC and was approved by the Royal Perth Hospital Ethics Committee. All volunteers gave informed written consent prior to participation.

### Subjects

125 volunteers were recruited through newspaper advertisements and flyers. Inclusion criteria were: male, Caucasian, non-smokers, BMI between 19 and 25, no hospital admissions within the preceding 5 years, no underlying chronic medical conditions and not using any topical or systemic medication likely to impact on the skin. (See additional data).

### Sample Collection

3 mm diameter full thickness punch biopsies were obtained from non sun-exposed lower back, approximately 5 cm lateral to the vertebral column, following subcutaneous injection of 1% lignocaine. Samples were immediately placed in an RNA stabilization agent (RNAlater, Ambion) and stored at 4 °C until processing.

### RNA extraction

Samples were dissected with scalpel in 0.5 ml of TRIzol reagent (Invitrogen) then homogenized on ice using a hand-held rotor-stator homogenizer (TissueRuptor, Qiagen) with disposable probes, in 10 s bursts for a total of 60 s. Samples were then processed with a standard TRIzol/chloroform and RNA Easy kit (Qiagen) purification protocol as per the manufacturer’s instructions. Quantity and quality of RNA was determined using an Agilent 2100 BioAnalyser (Agilent Technologies). Samples were discarded if the RNA integrity number (RIN) was <7.5, or <2 ug of RNA was obtained.

### Processing, Hybridization and Quality Control

Samples were prepared using the GeneChip Whole Transcript Sense Target Labeling Assay (Affymetrix) as per manufacturer’s instructions. 5.5 μg of fragmented, biotinylated, single stranded DNA was then hybridized to an Affymetrix Exon ST 1.0 Array for 17 hours at 45 °C and 60 rpm. Arrays were washed, stained and scanned on an Affymetrix Genechip Scanner 3000 as per the Affymetrix protocol. After scanning, CEL files were manually assessed for grid alignment and the absence of scratches and bubbles. Quality control was assessed using Affymetrix Expression Console software as per the manufacturer’s whitepaper on Quality Assessment of Exon and Gene 1.0 St Arrays. To minimize technical error, arrays were run in large batches consisting of a random distribution of samples across all age groups.

### Microarray Analysis

We focused our analysis on the approximately 230, 000 “core” exon probe sets that map to approximately 17,800 core genes derived from RefSeq and full-length GenBank mRNAs. Gene-level expression values were calculated using the RMA algorithm as implemented in Expression Console v1.1.1 (Affymetrix). Using the OneChannelGUI^40^ package in R Bioconductor, probe sets were filtered for reliable expression based upon the expression values of intron control probe sets and removal of cross hybridizing probe sets. Lastly, to generate a list of genes demonstrating the greatest level of change, an inter-quartile range filter was applied. This generated a list of 9333 genes which were used for the remainder of the analysis.

Subjects were grouped into 5 different age groups (additional data) and analyzed using limma^41^ and significance analysis of microarray (SAM)^42^, to determine differential expression of genes between age groups. 3885 genes were identified as significant based upon the overlap of P < 0.01 with limma analysis and a median FDR <0.05 with SAM. An analysis of variance (ANOVA) on age groups and a linear regression model (data not shown) identified a similar group of genes that change with age.

Gene Ontology analysis of the differentially expressed genes was conducted using the GOstats^43^ package in R Bioconductor, with P < 0.0001 treated as significant. Gene Set Enrichment Analysis^44^ (GSEA) was performed using the standard settings for a pre-ranked list analysis.

### Data visualization

For the purpose of data visualization, raw cel files were read into Genespring GX 10 (Agilent technologies), normalized with GC-RMA and median centered. Genes previously identified as differentially expressed between various age groups, were mapped across all five age groups.

### Interaction networks

Interaction networks were created for each list of differentially expressed genes in GeneSpring GX 10 using a database of relations between biological molecules and processes extracted from Medline and other public repositories through a natural language programming (NLP) algorithm. Networks were constructed to identify common factors involved in the following interaction types; expression, binding, regulation, promoter binding, transport, metabolism, or protein modification. Interactions were limited to high confidence relationships (NLP score 7 and above) and expanded to include nearest neighbor relationships. Networks were then limited to the largest observed clusters, to highlight common factors interacting with the identified genes. Extracted relations were imported into Cytoscape^45^, for production of final networks.

### Interaction Pathways

Pathway Studio Mammal^®^ (Elsevier) was used to explore the biological pathway changes in the microarray data. To look for common regulators, the top 1000 genes sorted by unadjusted p-value were input into the Pathway Studio. The ‘build pathway’ option was selected, after which the algorithm ‘find common regulators’ was selected and the minimum number of connections set to 200. The entity was then set to ‘protein’, and all types of relations were set.

### Heatmap construction

Heatmaps were generated using up and down regulated genes and the inbuilt heatmap function in R Studio^45^. Genes with a p value p < 0.01 and a 5% False discovery rate were used for all heatmaps. Patients were sorted by groups and individuals respectively.

### Comparison of previous microarray studies of human aging

Previous microarray studies of human aging were classified as defining “young” as between 18–29 or 30–45 years of age based upon the age of the youngest individuals in the study. Where studies had “young” individuals in both the 18–29 and 30–45 age groups they were assigned one of the two groups only if the vast majority of members of the group were within either the 18–29 or 30–45 age groups. For example, in Lu *et al*.^16^, “young” was defined as <43 years of age, with individuals aged 26–42, however the average age fell between 30–45 and was over 10 years greater than the average age of our 18–29 year old age group and hence was categorized as defining “young” as aged 30–45. Individual gene sets were constructed from the lists of genes identified as significantly changing with age in these studies. We then used GSEA to determine the level of enrichment of these gene sets within data obtained from skin.

Genes identified as changing with age in previous studies were mapped by their expression in skin across the lifespan. Unpaired, two-tailed t-tests in excel were used for direct comparison of the proportion of genes changing in expression between age-groups in studies identifying “young” as 18–29 vs. 30–45.

### Meta-analysis of previous microarray studies of human aging

Raw data from previous microarray studies was obtained from publically available microarray repositories. Raw data was converted to Affymetrix Human Transcriptome U133 Plus 2.0 data using best match probe sets. Data was normalized using RMA and analyzed using either two-sample t-tests (looking for differential gene expression between two age groups) or linear regression (looking for a correlation between gene expression and age over all age groups), using R version 2.12^46^. In each case the data was analyzed separately for each of the 9 cell types available, with a p-value calculated for each cell type. Genes were regarded as differentially expressed within an individual cell type if they had a P < 0.01. We then calculated the number of individual cell types per gene with a P < 0.01 that would be required such that a maximum of 5 false positives genes would be identified, All genes that had at least this number of individual cell types with a P < 0.01 were classified as differentially expressed.

Previous studies were individually assessed for the presence/absence of a transient period of transcriptome variability at age 30–45. Raw data from previous studies were separated into the same age groups used in our original analysis, normalized with RMA and then assessed for differential expression as compared to the 18–29 age-group using a P < 0.01 with limma.

### Quantitative real-time PCR validation

The Human Universal Probe Library system (Roche) was used for RT qPCR with βeta 2 micoglobulin and Succinate dehydrogenase complex, subunit A, chosen using the GeNorm algorithm^47^, serving as endogenous controls. RNA from a minimum of 5 individuals from each age group tested (18–29 and 30–45) was reverse transcribed using SuperScript II^TM^ RNase H reverse transcriptase (Invitrogen) as per manufacturer’s guidelines. cDNA was diluted 1 in 40. Expression levels of 12 genes were tested for each individual RNA sample, 8 genes identified as upregulated in the 30–45 age group (RB1, TGFB1, REL, APAF1, CHUK, FAS, MAPK, NFKB) and 4 genes identified as downregulated at age 30–45 (COL1A1, ELN, COL3A1 and IKBKG). In addition 9 different genes were tested for levels of expression in the youngest (18–29) and oldest (>70) age groups. At least 5 individuals were used in each age group for each gene. No samples were pooled and each reaction for each gene/sample was performed in triplicate. Each reaction comprised of 4 μL diluted cDNA, 5 μL 2 × LightCycler 480 Probes Master (Roche), 0.1 μL each forward and reverse primer (20 μM), 0.1 μL Probe (10 μM) (Roche), and 0.7 μL of water. Amplifications were run on a LightCycler^®^ 480 real time PCR machine, with thermal cycling conditions: 95 °C for 5 min, 50 cycles of denaturation at 95 °C for 10 s and annealing and extension at 60 °C for 30 s. Amplification efficiencies were close to 100% for all assays. The amounts of target genes expressed in a sample were normalized to the average of the two endogenous controls. Statistical significance was determined using two-tailed t-tests for each probe in excel.

## Additional Information

**How to cite this article**: Haustead, D. J. *et al*. Transcriptome analysis of human ageing in male skin shows mid-life period of variability and central role of NF-κB. *Sci. Rep*. **6**, 26846; doi: 10.1038/srep26846 (2016).

## Supplementary Material

Supplementary Information

## Figures and Tables

**Table 1:** Upregulated gene sets from previous microarray studies- Enrichment in skin from ages 18–29 to 70+.

Tissue	Study	Gender	Age of Youngest Individuals	Size	Enrichment*	Q Value
Kidney General	Rodwell *et al*.^13^	Mixed	30–45	164	2.43	0.000
Kidney Specific	Rodwell *et al*.^13^	Mixed	30–45	88	2.21	0.000
Brain	Berchtold *et al*.^12^	Male	30–45	862	1.87	0.000
Muscle	Zahn *et al*.^15^	Mixed	18–29	26	1.83	0.000
Brain	Berchtold *et al*.^12^	Female	30–45	515	1.81	0.000
Cortex (Brain)	Lu *et al*.^16^	Mixed	30–45	137	1.63	0.006
Muscle	Welle *et al*.^14^	Male	1829	76	1.47	0.026

Gene sets were constructed from lists of genes identified as significantly increasing in expression with age in previous studies. Using GSEA these gene sets were then assessed for their level of enrichment within expression data from human skin.

^*^A score of 0 indicates no enrichment.
