# Supplementary material for: Transcriptome analysis of human ageing in male skin shows mid-life period of variability and central role of NF-κB
Source: Sci Rep. 2016 May 27;6:26846. doi: 10.1038/srep26846 (PMC4882522; doi:10.1038/srep26846)
Supplement: Supplementary Information [file srep26846-s1.pdf]

Transcriptome analysis of human ageing in male skin shows mid-life period of variability and central role of NF-κB

Daniel J. Haustead[1,2,3] Andrew Stevenson[1,4] Vishal Saxena[5], Fiona Marriage[3], Martin Firth[2], Robyn Silla[1,6], Lisa Martin[1,6], Katharine F. Adcroft[1,4], Suzanne Rea[1,4,6], Philip J. Day[3], Philip E. Melton[7], Fiona M. Wood[1,2,4,6], Mark W. Fear*[1,4]

[1]The Fiona Wood Foundation, Perth, WA, Australia 6000. [2]Faculty of Medicine, Dentistry and Health Sciences, University of Western Australia, Crawley WA, Australia 6009. [3]Faculty of Medicine and Health Sciences, University of Manchester, Manchester, UK, M1 7DN. [4]Burn Injury Research Unit, School of Surgery, University of Western Australia, Crawley WA, Australia 6009. [5]Department of Mechanical Engineering, Massachusetts Institute of Technology, Cambridge, Massachusetts, USA 02139. [6]Burns Service of Western Australia, Royal Perth Hospital and Princess Margaret Hospital, WA, Australia 6000. [7]Centre for genetic origins of health and disease, University of Western Australia, Crawley, WA, Australia, 6009

a

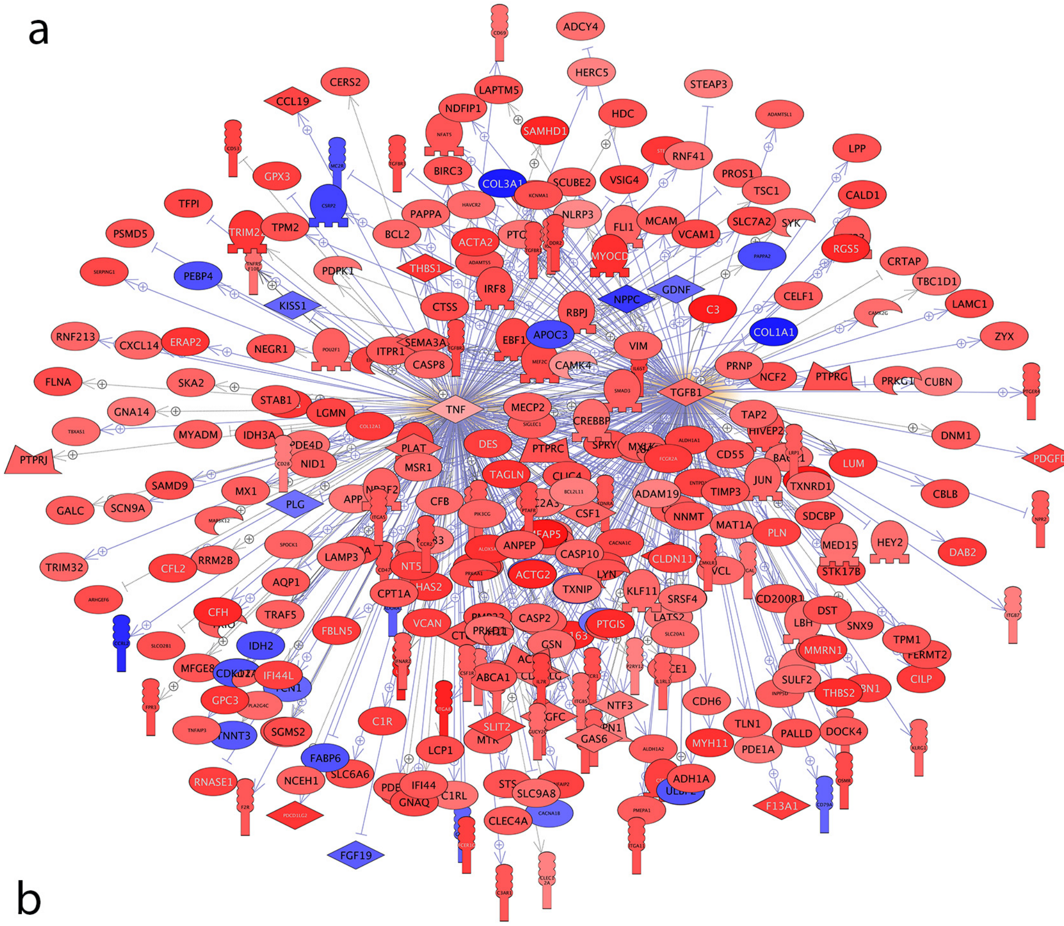

ADCY4
HERC5
CERS2
CCL19
LAPTM5
NDFIP1
SAMHD1
HDC
STEAP3
LPP
GPX3
TFPI
PSMD5
TRIM22
TPM2
BIRC3
COL3A1
SCUBE2
VSIG4
RNF41
PROS1
TSC1
CALD1
PAPPA
BCL2
NLRP3
FLI1
MCAM
VCAM1
SLC7A2
SYK
RGS5
CRTAP
TBC1D1
PEBP4
PDPK1
ACTA2
THBS1
MYOCD
IRF8
NPPC
GDNF
C3
CELF1
LAMC1
KISS1
CTSS
RBPJ
COL1A1
ZYX
RNF213
CXCL14
ERAP2
NEGR1
ITPR1
SEMA3A
EBF1
APOC3
VIM
CASP8
CAMK4
PRNP
NCF2
PTPRG
PRKG1
CUBN
FLNA
SKA2
STAB1
TNF
MECP2
TGFB1
GNA14
LGMN
CREBBP
TAP2
HIVEP2
DNM1
MYADM
IDH3A
PDE4D
SPRY
CD55
PDGFD
PTPRJ
NID1
PLAT
MSR1
DES
PTPRC
LUM
TXNRD1
CLIC4
TIMP3
JUN
CBLB
SAMD9
MX1
TAGLN
ADAM19
GALC
SCN9A
PLG
APP
CFB
CSF1
NNMT
SDCBP
MAT1A
PLN
HEY2
MED15
DAB2
TRIM32
RRM2B
CFL2
LAMP3
ANPEP
CLDN11
STK17B
AQP1
CASP10
ACTG2
LYN
VCL
TXNIP
KLF11
SRSF4
CD200R1
CFH
TRAF5
FBLN5
VCAN
CASP2
PTGIS
DST
LBH
SNX9
TPM1
FERMT2
IDH2
PRKD1
GSN
MMRN1
CILP
MFGE8
IFI44L
ABCA1
SULF2
THBS2
GPC3
C1R
NTF3
CDH6
TNNT3
SGMS2
SLIT2
GAS6
TLN1
DOCK4
LCP1
MYH11
PDE1A
PALLD
FABP6
MTR
RNASE1
NCEH1
SLC6A6
IFI44
GNAQ
C1RL
STS
SLC9A8
ADH1A
F13A1
CLEC4A
FGF19

b

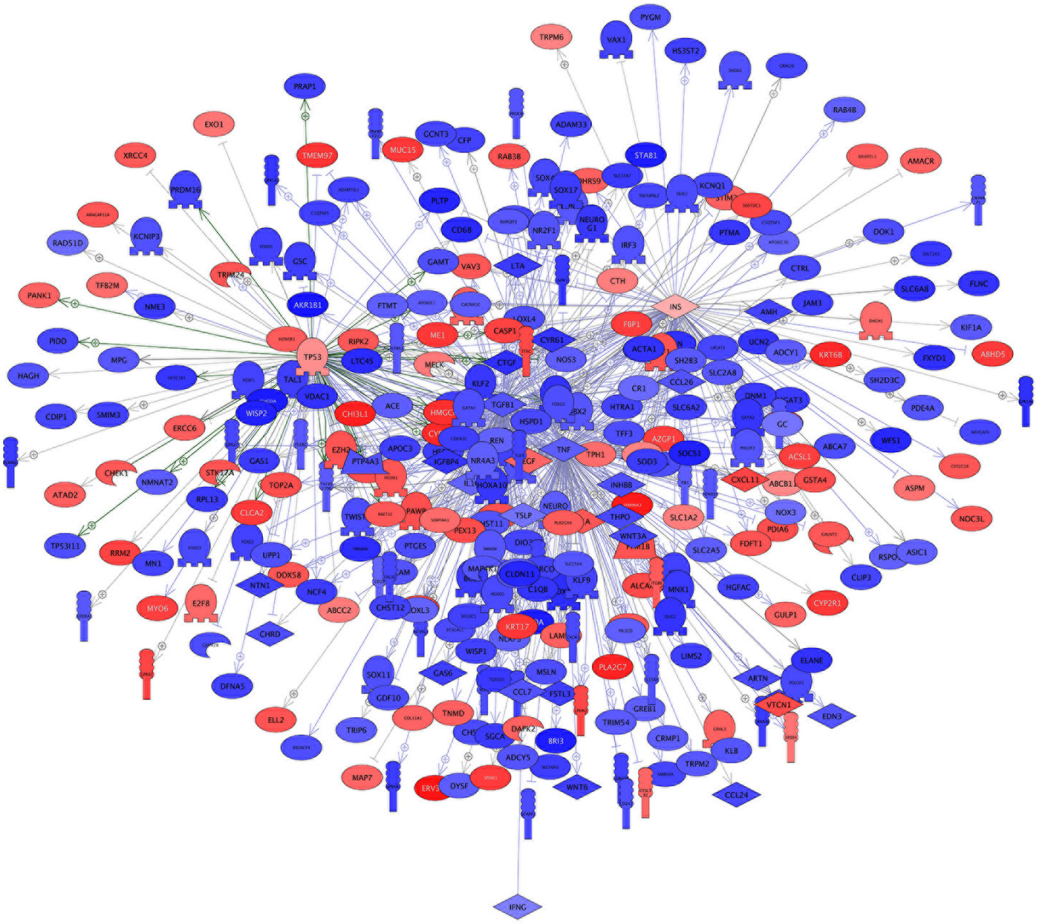

TRPM6
VAX1
PYGM
HS3ST2
PRAP1
EXO1
XRCC4
TMEM97
ADAM33
MUC15
GCNT3
CFP
RAB3B
STAB1
RAB4B
AMACR
PRDM16
KCNIP3
RAD51D
PLTP
CD68
NR2F1
IRF3
KCNQ1
PTMA
DOK1
GSC
GAMT
VAV3
LTA
CTH
CTRL
SLC6A8
FLNC
PANK1
TFB2M
NME3
AKR1B1
FTMT
INS
AMH
JAM3
KIF1A
PIDD
MPG
TP53
ME1
CASP1
CYR61
FBP1
ACTA1
ADCY1
KRT68
FXYD3
ABHD5
HAGH
LTC4S
RIPK2
LOXL4
CTGF
NOS3
SH2B3
SLC2A8
UCN2
SH2D3C
CDIP1
SMIM3
VDAC1
WISP2
KLF2
TGFB1
HSPD1
CR1
HTRA1
CCL26
SLC6A2
DNM1
PDE4A
ERCC6
ACE
APOC3
REN
TNF
TFF3
TPH1
AZGP1
SOCS1
GC
ABCA7
WFS1
CHEK1
STK17A
GAS1
EZH2
PTP4A3
IGFBP4
NR4A3
SOD3
ACSL1
CXCL11
GSTA4
ATAD2
NMNAT2
TOP2A
HOXA10
INHBB
ABCB1
ASPM
RPL13
CLCA2
TWIST1
PEX13
TSLP
THPO
SLC1A2
NOX3
FDFT1
PDIA6
NOC3L
TP53I11
RRM2
MN1
UPP1
PTGES
DIO2
WNT3A
SLC2A5
RSPO
ASIC1
CLDN11
KLF9
MNX1
HGFAC
CLIP3
NTN1
DDX58
NCF4
CHST12
C1QB
CYP2R1
MYO6
E2F8
ABCC2
KRT17
GULP1
CHRD
WISP1
LIMS2
DFNA5
SOX11
GAS6
MSLN
PLA2G7
ELANE
GDF10
CCL7
FSTL3
ARTN
VTCN1
EDN3
ELL2
TNMD
TRIM54
GREB1
TRIP6
DAPK2
BRI3
CRMP1
KL8
ADCY5
TRPM2
MAP7
ERV3
DYSF
WNT6
CCL24
IFNG

Figure S1

a Pathway showing common regulators with >100 interacting proteins in the pathway list for the eldest versus youngest age groups comparison.

b Pathway showing common regulators with >100 interacting proteins in the pathway list for the 30-45 age group versus all other age groups.

Red indicates increased gene expression, blue indicates decreased gene expression in the eldest (a) and mid-life (b) groups compared to other age groups.

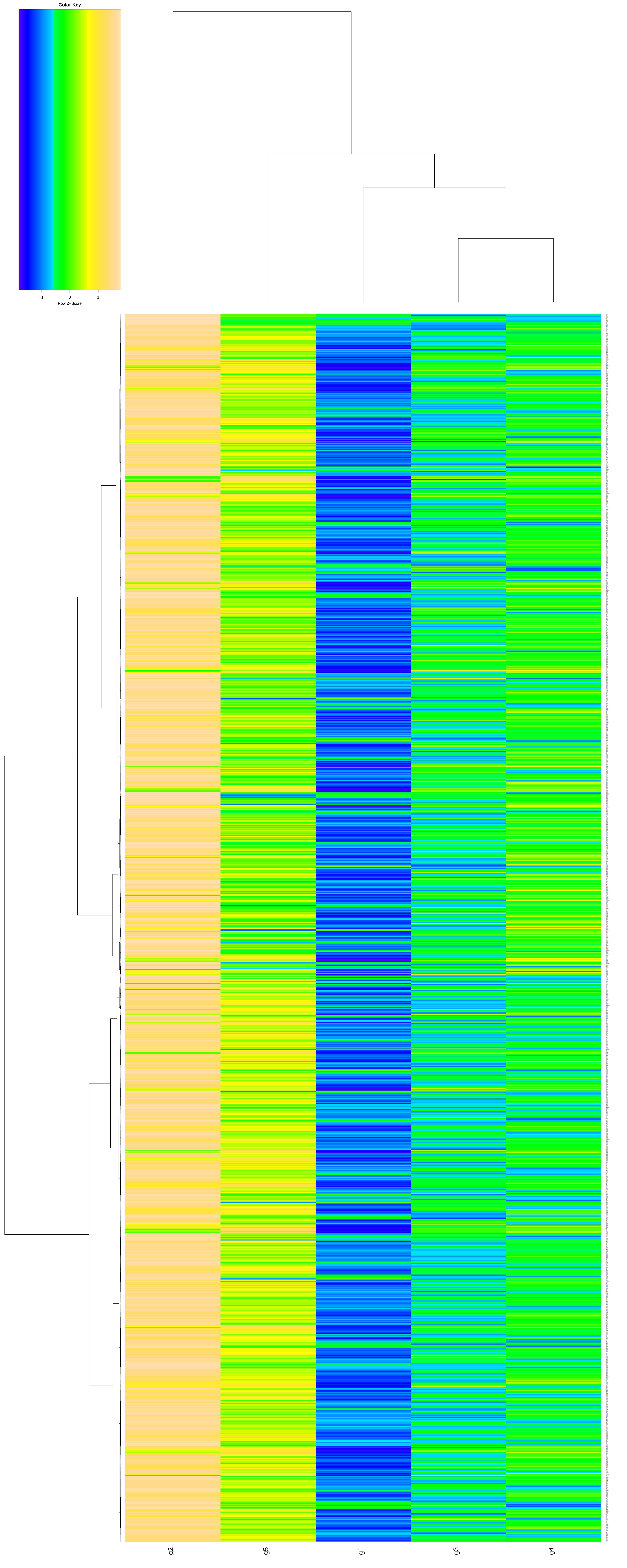

Color Key
−1
0
1
Row Z−Score
g2
g5
g1
g3
g4

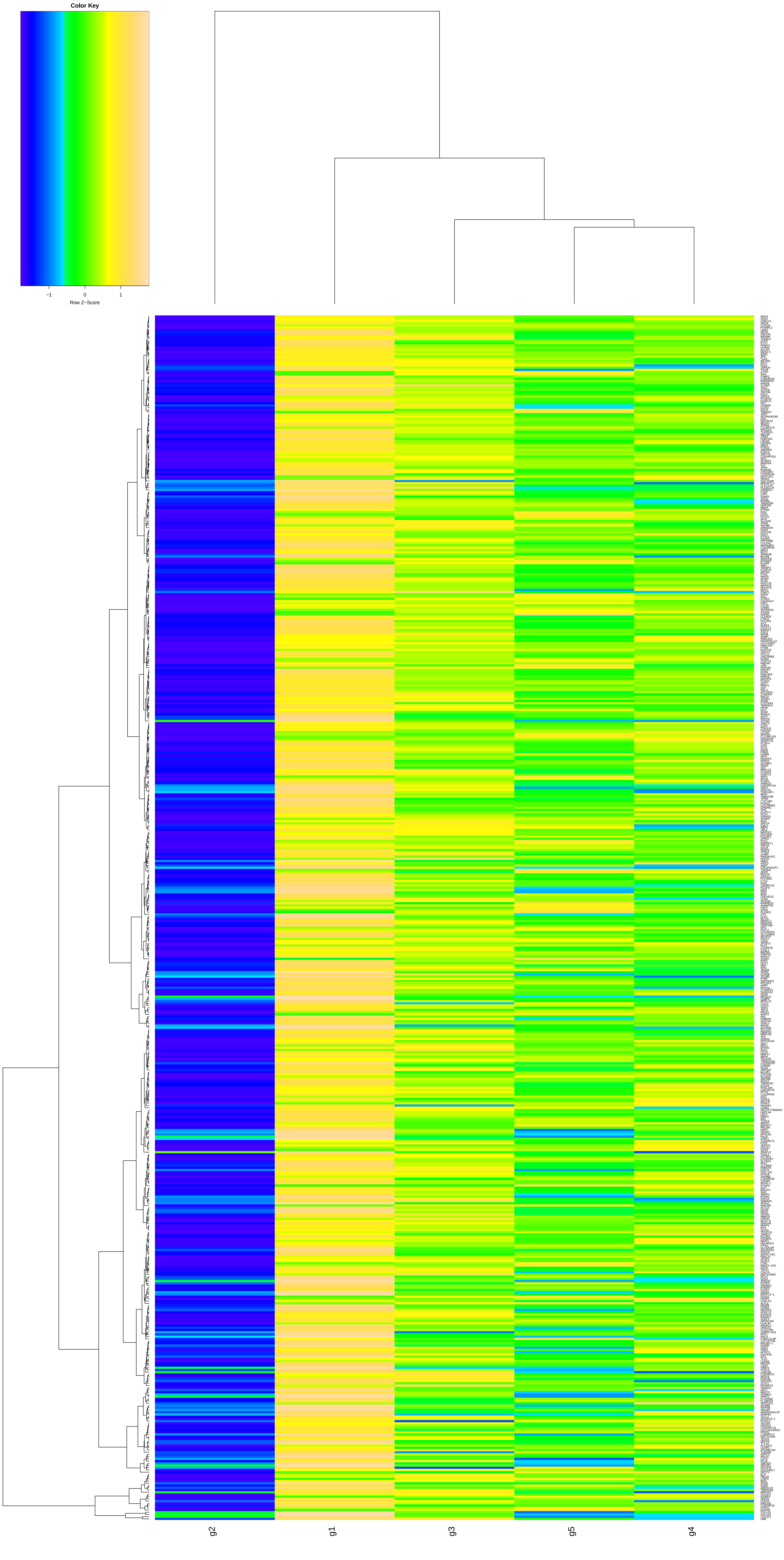
Color Key
−1
0
1
Row Z−Score
g2
g1
g3
g5
g4

Figure S2

a Heatmap of genes upregulated in the 30-45 age group compared to other age groups shown as a group comparison

b Heatmap of genes downregulated in the 30-45 age group compared to other age groups shown as a group comparison

g1 18-29, g2 30-45, g3 46-60, g4 61-75, g5 >75 age groups.

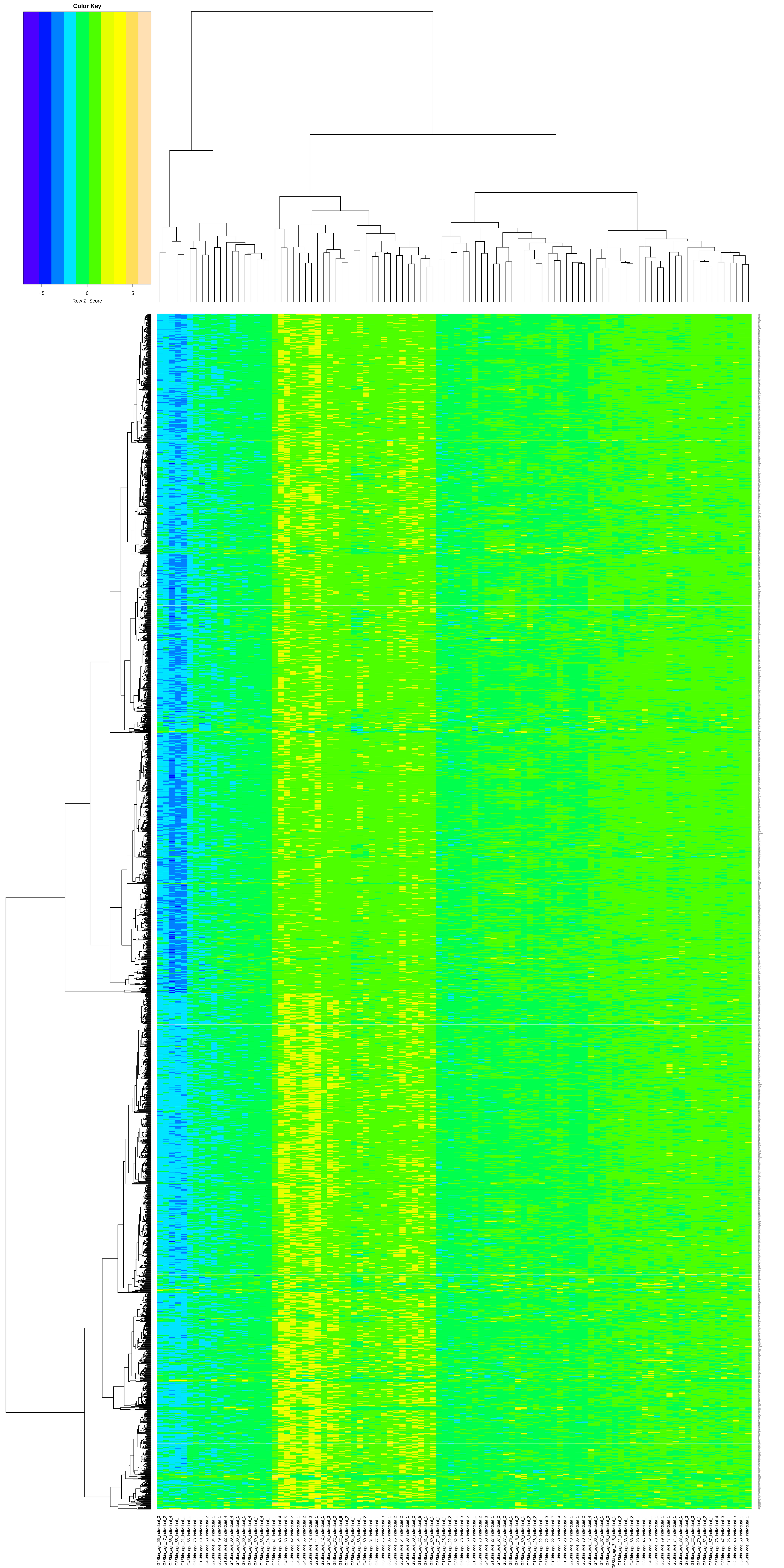
Color Key
−5
0
5
Row Z−Score

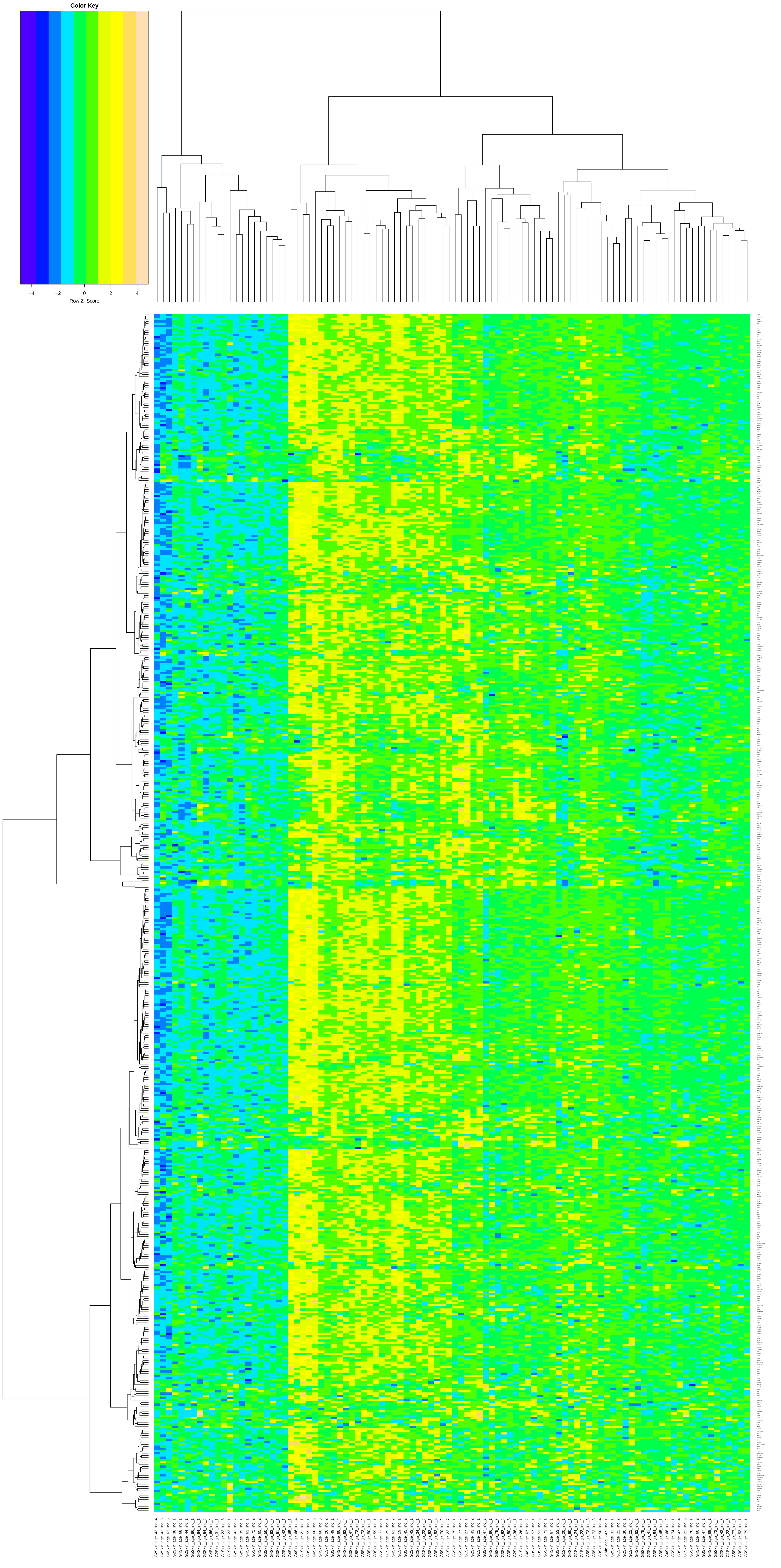
Color Key
−4
−2
0
2
4
Row Z−Score

Figure S3

a Heatmap of genes upregulated in the 30-45 age group compared to other age groups showing individual samples.

b Heatmap of genes downregulated in the 30-45 age group compared to other age groups showing individual samples.

Age	Yrs in Aus	Past Med Hx	Medications	Cur Smoker	Years post quitting	Amount smoked (pack years)	Fam Hx Skin Ca
19	13	No	Nil	No	NA	NA	Nil
20	20	No	Nil	No	NA	NA	Nil
21	21	Hayfever/Allergies	Nil	No	NA	NA	Nil
21	21	No	Nil	No	NA	NA	Nil
22	22	No	Nil	No	NA	NA	Nil
22	22	No	Nil	No	NA	NA	Nil
22	20	No	Nil	No	NA	NA	Nil
22	22	No	Nil	No	NA	NA	Nil
22	22	Migraines	Nil	No	NA	NA	Nil
22	22	migraines	Nil	No	NA	NA	Nil
22	20	No	Nil	No	NA	NA	Nil
23	23	No	Nil	No	NA	NA	Nil
23	23	No	Nil	No	NA	NA	Nil
24	24	No	Nil	No	NA	NA	Nil
25	25	No	Nil	No	2 years	NA	Nil
26	26	No	Nil	No	NA	NA	Nil
26	26	No	Nil	No	NA	NA	Nil
27	27	No	Nil	No	NA	NA	Nil
30	30	Asthma	Formoterol, Budesonide	No	NA	NA	Nil
31	31	No	Nil	No	NA	NA	Nil
33	33	Deaf in right ear	Nil	No	NA	NA	Nil
34	34	No	Nil	No	NA	NA	Father-benign
36	36	No	Nil	No	NA	NA	Nil
38	38	No	Nil	No	NA	NA	Nil
39	39	Hypertension	Nil	No	6 years	1	Nil
41	30	No	Nil	No	NA	NA	Nil
42	21	No	Nil	No	NA	NA	Nil
42	42	No	Nil	No	2 years	3	Nil
42	42	No	Nil	No	NA	NA	Nil
43	43	No	Nil	No	NA	NA	Nil
43	43	No	Nil	No	NA	NA	Nil
43	43	No	Nil	No	NA	NA	Nil
43	43	No	Nil	No	NA	NA	Nil
44	20	No	Nil	No	NA	NA	Nil
45	45	No	Steroid Creams	No	NA	NA	Nil
45	45	No	Nil	No	NA	NA	Nil
47	8	No	Nil	No	7 years	NA	Mother-benign
47	47	No	Nil	No	NA	NA	Nil

47	34	No	Multi vitamins	No	NA	NA	Nil
47	47	GORD	Nil	No	NA	NA	Nil
49	30	No	Nil	No	NA	NA	Nil
50	50	No	Fish oils	No	30 years	12	Father- benign
50	50	No	Nil	No	35 years	NA	Nil
50	20	No	Nil	No	NA	NA	Nil
50	18	No	Nil	No	NA	NA	Nil
51	51	No	Nil	No	23 years	NA	Nil
52	52	No	Nil	No	NA	NA	Mother
52	15	No	Nil	No	NA	NA	Nil
53	53	No	Nil	No	20 years	5	Father- benign
53	53	No	Multi vitamins	No	NA	NA	Nil
53	25	No	Nil	No	NA	NA	Nil
53	53	No	Nil	No	NA	NA	Nil
54	54	No	Multi vitamins	No	30 years	NA	Nil
54	54	No	Nil	No	NA	NA	Nil
55	55	No	Nil	No	NA	NA	Nil
57	57	Depression	Citalopram	No	12 years	30	Nil
59	59	No	Nil	No	NA	NA	Nil
60	60	No	Nil	No	NA	NA	Father
60	48	No	Nil	No	15 years	5	Nil
60	60	No	Nil	No	NA	NA	Nil
60	60	No	Nil	No	NA	NA	Nil
62	39	No	Nil	No	NA	NA	Nil
63	63	No	Nil	No	NA	NA	Nil
63	63	No	Nil	No	NA	NA	Nil
63	63	No	Nil	No	20 years	<1	Nil
63	24	No	Nil	No	NA	NA	Nil
63	63	Burn to R leg, cholecystitis 1992, pneumonia 1968, appendicitis 1960	Omeprazole	No	15 years	NA	Nil
64	64	No	Nil	No	NA	NA	Nil
65	65	No	Nil	No	NA	NA	Nil
65	65	No	Multi vitamins	No	30 years	<1	Nil
66	40	Dupuytren's contracture L hand, BPH	Nil	No	NA	NA	Nil
66	66	No	Nil	No	23 years	NA	Nil
66	66	No	Nil	No	NA	NA	Nil
66	66	No	Atorvastatin, Ramipril 10mg	No	NA	NA	Nil
67	67	Asbestosis	Multi vitamins	No	45 years	<1	Nil

67	67	Cancer- right kidney (previously)	Irbesartan	No	40 years	5	Nil
68	13	No	Multi vitamins	No	40 years	Jan-04	Nil
68	36	No	Nil	No	35 years	17	Nil
69	40	No	Nil	No	NA	NA	Nil
70	70	No	Nil	No	NA	NA	Nil
70	70	SVT	Prazosin, Multi vitamins	No	NA	NA	Nil
72	72	Gout, prostate cancer (previously)	Multi vitamins	No	15 years	10	Nil
72	10	No	Nil	No	NA	NA	Nil
72	72	No	Irbesartan, Prazosin, Multi vitamins	No	NA	NA	Nil
73	73	No	Nil	No	NA	NA	Nil
73	37	No	Nil	No	NA	NA	Nil
73	73	No	Irbesartan, Atorvastatin, Amlodipine	No	25 years	10	Nil
73	73	Gout	Allopurinol, Multi vitamins	No	NA	NA	Nil
74	27	No	Nil	No	30 years	NA	Nil
75	75	No	Nil	No	NA	NA	Nil
75	29	Eczema, diptheria as child	Nil	No	NA	NA	Nil
75	44	No	Nil	No	NA	NA	Nil
75	75	No	Ramipril, Atorvastatin	No	NA	NA	Nil
77	55	BPH	Perindopril, tamsulosin	No	30 years	5	Nil
77	77	No (cataract 07, diptheria 1934, tonsillectomy)	Nil	No	NA	NA	Nil

Supplementary data table 1. Age, medication, smoking and skin cancer history of volunteers participating in the study

Symbol	Fold Change	P.Value
CACNA1C	1.44	2.27E-06
TMEM176B	1.55	4.06E-06
CASQ2	1.94	6.90E-06
THBS2	1.76	7.91E-06
GALNTL2	2.21	1.13E-05
NOX4	1.52	1.14E-05
CLDN11	1.52	1.77E-05
C3	2.06	2.01E-05
ITGA8	1.92	3.25E-05
CD163	1.85	4.67E-05
LMO3	1.66	5.60E-05
C2	1.38	5.97E-05
TAGLN	1.69	6.65E-05
CP	2.14	7.66E-05
PTGIS	1.80	7.92E-05
VIT	1.82	9.07E-05
ACTA2	1.63	9.69E-05
ABCA9	2.02	1.01E-04
LEPR	1.87	1.24E-04
ITK	1.36	1.35E-04
PRSS12	1.41	1.37E-04
STMN2	1.92	1.73E-04
C1S	1.73	1.73E-04
IL10RA	1.50	1.77E-04
TNFAIP2	1.48	2.01E-04
ALOX5AP	1.71	2.31E-04
LAMA2	1.71	2.54E-04
PRUNE2	1.59	2.69E-04
SAMHD1	1.85	4.48E-04
ACTC1	2.52	4.50E-04
TLR4	1.58	4.77E-04
MYOCD	1.71	5.21E-04
MFAP4	1.90	5.25E-04
GPR133	1.41	5.29E-04
CD209	1.55	5.46E-04
TMEM176A	1.53	5.55E-04
SYT11	1.41	6.26E-04
PCDHB3	1.51	6.52E-04
RBPMS	1.41	6.87E-04
LMOD1	1.45	6.92E-04
RGL1	1.48	7.13E-04
CPE	1.72	7.25E-04

CD84	1.42	7.57E-04
VSIG4	1.49	7.85E-04
ALDH1B1	1.43	8.60E-04
MS4A6A	1.60	8.70E-04
MFAP5	2.16	9.01E-04
RBMS3	1.59	9.22E-04
ACTG2	1.92	9.61E-04
RPL24	1.69	9.95E-04
PDCD1LG2	1.63	9.98E-04
NCKAP1L	1.49	1.03E-03
SLIT3	1.61	1.13E-03
FLNA	1.38	1.15E-03
VCAM1	1.42	1.17E-03
APOBEC3C	1.51	1.18E-03
TMEM71	1.51	1.24E-03
GLIPR1	1.92	1.34E-03
P2RX1	1.40	1.39E-03
SRPX2	1.51	1.41E-03
GRK5	1.29	1.43E-03
PGM5	1.41	1.48E-03
C7	1.63	1.54E-03
PLEKHH2	1.59	1.56E-03
TMOD1	1.31	1.68E-03
SMOC2	1.62	1.69E-03
FAT	1.49	1.82E-03
LTBP2	1.34	1.83E-03
KLF7	1.33	1.88E-03
PRUNE2	1.66	1.89E-03
GPC3	1.63	1.94E-03
REEP3	1.64	1.96E-03
FAM133B	1.52	2.06E-03
FCGR2A	1.56	2.07E-03
FAM69A	1.37	2.14E-03
ENTPD1	1.48	2.26E-03
LHFP	1.41	2.33E-03
C1orf162	1.54	2.36E-03
C10orf64	1.29	2.43E-03
SLCO2B1	1.36	2.56E-03
TMEM173	1.29	2.60E-03
MX2	1.44	2.62E-03
MYH11	1.69	2.84E-03
SLC6A6	1.38	2.86E-03
RNASE1	1.52	3.02E-03
RGS5	1.84	3.08E-03
DPYSL3	1.39	3.28E-03
ISLR	1.65	3.29E-03
KANK2	1.35	3.35E-03

C1R	1.54	3.43E-03
PCDHB16	1.51	3.45E-03
CTGF	1.35	3.52E-03
ELK3	1.35	3.57E-03
ABCA6	1.69	3.58E-03
CCL13	1.75	3.72E-03
BHMT2	1.33	3.72E-03
THBS1	1.66	3.97E-03
PRRX1	1.52	4.01E-03
CCR1	1.37	4.09E-03
CFL2	1.52	4.23E-03
COLEC12	1.33	4.29E-03
FBLN5	1.58	4.38E-03
PLSCR4	1.44	4.45E-03
DOCK2	1.37	4.46E-03
CMKLR1	1.31	4.52E-03
FOLR2	1.43	4.57E-03
PLEK	1.40	4.58E-03
PLN	1.50	4.60E-03
ST3GAL5	1.35	4.62E-03
CSRP1	1.50	4.66E-03
PTPRG	1.36	4.75E-03
STAB1	1.35	4.83E-03
MS4A4A	1.68	4.84E-03
ALDH1A1	1.49	4.85E-03
C1QA	1.43	4.94E-03
IL7R	1.49	5.05E-03
MMRN1	1.64	5.07E-03
IRF8	1.37	5.11E-03
CFH	1.82	5.22E-03
CYBB	1.63	5.28E-03
FAM70A	1.24	5.38E-03
PRG4	2.30	5.42E-03
CCBE1	1.40	5.43E-03
SORBS1	1.40	5.50E-03
UBLCP1	1.58	5.59E-03
ITGA5	1.31	5.68E-03
SPRED2	1.33	5.89E-03
TNS1	1.40	5.91E-03
TRIM22	1.62	6.05E-03
MAOB	1.34	6.23E-03
FHL1	1.43	6.28E-03
AOX1	1.56	6.31E-03
SPRY1	1.37	6.37E-03
TGFBR1	1.39	6.39E-03
CNN1	1.47	6.43E-03
FCER1G	1.50	6.46E-03

C1QTNF7	1.44	6.47E-03
PDGFD	1.51	6.55E-03
TGFBR2	1.43	6.59E-03
ANK2	1.42	6.65E-03
TBC1D2B	1.40	6.70E-03
HDC	1.36	6.80E-03
PMP22	1.43	6.83E-03
CILP	1.58	6.96E-03
FPR3	1.37	7.11E-03
DPYSL2	1.35	7.30E-03
CHRDL1	1.63	7.34E-03
LAMC1	1.41	7.42E-03
TFPI	1.44	7.45E-03
LPP	1.35	7.56E-03
PSD3	1.56	7.56E-03
IGJ	2.88	7.71E-03
TIMP3	1.36	7.81E-03
RBPJ	1.31	7.92E-03
ZNFX1	1.34	7.95E-03
CDH11	1.31	8.24E-03
NRP1	1.39	8.24E-03
CD200R1	1.33	8.25E-03
XPNPEP2	1.30	8.34E-03
CYBRD1	1.43	8.54E-03
SIPA1L2	1.31	8.66E-03
TGFBR3	1.38	8.69E-03
VCAN	1.55	8.77E-03
MYLK	1.29	8.87E-03
PSCD4	1.27	8.99E-03
MOXD1	1.31	9.02E-03
MAP1B	1.44	9.21E-03
PDE3A	1.31	9.37E-03
STEAP4	1.62	9.42E-03
CTSO	1.39	9.44E-03
EVI2B	1.53	9.54E-03
COL12A1	1.57	9.59E-03
CBLB	1.36	9.60E-03
C1QC	1.45	9.83E-03

Supplementary data table 2 – Genes upregulated in oldest versus youngest age groups (>70 vs 18-29 years)

Name	GO Term Number	Enrichment(NES)	P.Value
Nuclear Part	0044428	4.11	0.00
Intracellular Transport	0046907	4.08	0.00
Rna Binding	0003723	3.96	0.00
Organelle Membrane	0031090	3.79	0.00
Cellular Localization	0051641	3.76	0.00
Establishment Of Cellular Localization	0051234	3.72	0.00
Mitochondrion	0005739	3.66	0.00
Regulation Of Transcription	0006355	3.58	0.00
Membrane Enclosed Lumen	0031974	3.58	0.00
Organelle Lumen	0043233	3.54	0.00
Transcription From Rna Polymerase Ii Promoter	0006368	3.49	0.00
Nuclear Lumen	0031981	3.39	0.00
Macromolecule Localization	0033036	3.28	0.00
Macromolecular Complex Assembly	0065003	3.19	0.00
Protein Localization	0008104	3.18	0.00
Cytoskeletal Protein Binding	0008092	3.17	0.00
Catabolic Process	0009056	3.17	0.00
Organelle Envelope	0031967	3.16	0.00
Regulation Of Rna Metabolic Process	0051252	3.16	0.00
Negative Regulation Of Transcription	0045892	3.15	0.00
Endoplasmic Reticulum	0005783	3.14	0.00
Cellular Component Assembly	0022607	3.13	0.00
Envelope	0005635	3.11	0.00
Structural Constituent Of Ribosome	0003735	3.1	0.00
Negative Regulation Of Transcription Dna Dependent	0045892	3.08	0.00
Cellular Catabolic Process	0044109	3.06	0.00
Regulation Of Transcription dna Dependent	0006355	3.06	0.00
Rna Processing	0006396	3.05	0.00
Negative Regulation Of Rna Metabolic Process	0051253	3.05	0.00
Golgi Apparatus	0005794	3.03	0.00
Identical Protein Binding	0042802	3.03	0.00
Endomembrane System	0012505	3	0.00
Protein Transport	0015031	2.98	0.00
Establishment Of Protein Localization	0045184	2.94	0.00
Vesicle Mediated Transport	0016192	2.93	0.00
Organelle Organization And Biogenesis	0006996	2.92	0.00
Translation	0006412	2.91	0.00
Rna Splicing	0008380	2.86	0.00
Protein Kinase Cascade	0035556	2.84	0.00

Transferase Activity Transferring Phosphorus Containing Groups	0016772	2.83	0.00
Outer Membrane	0019867	2.82	0.00
Biosynthetic Process	0009058	2.81	0.00
Negative Regulation Of Nucleobase nucleoside nucleotide And Nucleic Acid Metabolic Process	0045934	2.8	0.00
Nucleoplasm	0005654	2.79	0.00
Regulation Of Developmental Process	0050793	2.78	0.00
Programmed Cell Death	0012501	2.77	0.00
Regulation Of Apoptosis	0042981	2.77	0.00
Regulation Of Transcription From Rna Polymerase II Promoter	0006357	2.77	0.00
Apoptosis Go	0006915	2.72	0.00
Organelle Outer Membrane	0031968	2.72	0.00
Post Translational Protein Modification	0043687	2.72	0.00
Intracellular Protein Transport	0006886	2.71	0.00
Cellular Biosynthetic Process	0044249	2.69	0.00
Cytosol	0005829	2.68	0.00
Regulation Of Programmed Cell Death	0043067	2.67	0.00
Transcription Activator Activity	0016563	2.67	0.00
Negative Regulation Of Cellular Metabolic Process	0031324	2.67	0.00
Transcription Cofactor Activity	0003712	2.67	0.00
Negative Regulation Of Metabolic Process	0009892	2.66	0.00
I-kappaB kinase/NF-kappaB signaling	0007249	2.64	0.00
Transcription Factor Binding	0000989	2.63	0.00
Organic Acid Metabolic Process	0006082	2.6	0.00
Phosphotransferase Activity Alcohol Group As Acceptor	0016773	2.6	0.00
Kinase Activity	0016301	2.59	0.00
Protein N Terminus Binding	0047485	2.59	0.00
Carboxylic Acid Metabolic Process	0019752	2.58	0.00
Hydrolase Activity Acting On Acid Anhydrides	0016817	2.58	0.00
Pyrophosphatase Activity	0016462	2.55	0.00
Basement Membrane	0005604	2.54	0.00
Phosphorylation	0016310	2.53	0.00
Nucleoside Triphosphatase Activity	0017111	2.52	0.00
Cell Fraction	0000267	2.51	0.00
Golgi Apparatus Part	0044431	2.5	0.00
Regulation Of I Kappab Kinase Nf Kappab Cascade	0043122	2.5	0.00
Protein Targeting	0006605	2.5	0.00
Cytoskeleton	0005856	2.5	0.00

NES- normalised enrichment score (NES of 0 is no enrichment) **Supplementary data table 3 GSEA of positively enriched gene sets in oldest versus youngest age groups**

Term	GO Term Number	P.value
intracellular	0005622	3.50E-38
cell	0007049	4.80E-27
biopolymer metabolic process	0043170	4.50E-25
cellular macromolecule metabolic process	0044260	8.50E-22
ribonucleotide binding	0032553	2.80E-21
ATP binding	0005524	9.00E-21
macromolecule metabolic process	0043170	9.10E-21
adenyl ribonucleotide binding	0032559	3.80E-20
nucleus	0005634	5.30E-20
purine nucleotide binding	0017076	1.80E-19
nucleotide binding	0000166	1.30E-18
adenyl nucleotide binding	0030554	2.50E-18
organelle	0031090	4.20E-17
nucleobase, nucleoside, nucleotide and nucleic acid metabolic process	0055134	1.90E-15
intracellular membrane-bounded organelle	0043231	6.00E-14
transferase activity, transferring phosphorus-containing groups	0016772	2.70E-13
primary metabolic process	0044238	2.30E-12
protein serine/threonine kinase activity	0004674	2.30E-12
nuclear part	0044428	4.10E-12
nucleic acid binding	0003676	4.10E-12
ubiquitin-dependent protein catabolic process	0006511	1.00E-11
modification-dependent protein catabolic process	0019941	1.00E-11
cellular biopolymer catabolic process	0044265	1.00E-11
modification-dependent macromolecule catabolic process	0043632	1.00E-11
cellular protein catabolic process	0044257	1.00E-11
proteolysis involved in cellular protein catabolic process	0051603	1.00E-11
protein catabolic process	0030163	1.90E-11
nucleoplasm	0005654	2.20E-11
biopolymer catabolic process	0043285	3.70E-11
zinc ion binding	0008270	1.90E-10
nuclear lumen	0031981	2.70E-10
protein modification process	0036211	3.20E-10
ligase activity	0016874	3.60E-10
cellular metabolic process	0044237	4.40E-10
RNA metabolic process	0016070	5.70E-10
biopolymer modification	0043412	9.70E-10
phosphotransferase activity, alcohol group as acceptor	0016773	1.20E-09

kinase activity	0016301	3.40E-09
gene expression	0010467	4.30E-09
protein kinase activity	0004672	4.50E-09
intracellular organelle lumen	0070013	5.10E-09
post-translational protein modification	0043687	8.10E-09
protein metabolic process	0019538	8.30E-09
cellular protein metabolic process	0044267	8.40E-09
response to DNA damage stimulus	0006974	1.10E-08
helicase activity	0004386	2.30E-08
catalytic activity	0003824	2.50E-08
organelle lumen	0043233	2.60E-08
DNA repair	0006281	3.20E-08
membrane-enclosed lumen	0031974	3.40E-08
cellular response to DNA damage stimulus	0006974	4.70E-08
macromolecule biosynthetic process	0009059	5.40E-08
biopolymer biosynthetic process	0009059	5.70E-08
cellular response to stress	0033554	6.60E-08
cellular macromolecule biosynthetic process	0034645	6.60E-08
cellular macromolecule catabolic process	0044265	7.10E-08
protein amino acid phosphorylation	0006468	7.80E-08
ligase activity, forming carbon-nitrogen bonds	0016879	8.50E-08
macromolecule localization	0033036	8.70E-08
transition metal ion binding	0046914	9.40E-08
nucleoside-triphosphatase activity	0017111	1.10E-07
acid-amino acid ligase activity	0016881	1.20E-07
binding	0005488	1.30E-07
protein complex	0043234	1.90E-07
hydrolase activity, acting on acid anhydrides	0016817	2.10E-07
metabolic process	0008152	2.40E-07
pyrophosphatase activity	0016462	2.80E-07
organelle part	0044422	2.90E-07
ubiquitin thiolesterase activity	0004221	2.90E-07
macromolecule catabolic process	0009057	3.10E-07
ubiquitin-protein ligase activity	0061630	3.20E-07
protein localization	0008104	9.00E-07
protein transport	0015031	1.40E-06
establishment of protein localization	0045184	1.50E-06
ATP-dependent helicase activity	0008026	1.50E-06
GTPase activator activity	0005096	2.00E-06
cell cycle	0007049	2.30E-06
nucleoplasm part	0044451	2.90E-06

GTPase regulator activity	0030695	2.90E-06
cellular response to stimulus	0051716	3.00E-06
RNA binding	0003723	3.30E-06
ATPase activity	0016887	3.30E-06
Ubiquitin mediated proteolysis	0043161	4.10E-06
nucleobase, nucleoside, nucleotide and nucleic acid transport	0015931	5.40E-06
DNA metabolic process	0006259	6.10E-06
nucleic acid transport	0050657	6.40E-06
transcription	0003700	6.90E-06
phosphate metabolic process	0006796	7.10E-06
hydrolase activity, acting on ester bonds	0016788	8.10E-06
macromolecular complex	0032991	9.90E-06
hydrolase activity	0016787	1.20E-05
transferase activity	0016740	1.20E-05
DNA binding	0003677	1.40E-05
regulation of gene expression	0010468	1.50E-05
intracellular transport	0046907	1.90E-05
cellular process	0009987	2.00E-05
chromosome organization	0051276	2.10E-05
DNA replication	0006260	2.60E-05
cellular protein localization	0034613	2.60E-05
ATPase activity, coupled	0042623	2.80E-05
small conjugating protein ligase activity	0061659	2.90E-05
intracellular protein transport	0006886	3.00E-05
microtubule organizing center	0005815	3.30E-05
thiolester hydrolase activity	0016790	3.40E-05
non-membrane-bounded organelle	0043228	3.50E-05
mRNA transport	0051028	3.60E-05
nuclear pore	0005643	4.00E-05
centrosome	0005813	4.40E-05
cell division	0051301	4.50E-05
regulation of macromolecule biosynthetic process	0010556	5.30E-05
RNA Degredation	0000292	6.00E-05
regulation of small GTPase mediated signal transduction	0051056	6.70E-05
RNA biosynthetic process	0032774	6.70E-05
RNA processing	0006396	7.30E-05
phosphorylation	0016310	7.40E-05
regulation of nucleobase, nucleoside, nucleotide and nucleic acid metabolic process	0019219	7.60E-05
cellular localization	0051641	7.80E-05
protein modification by small protein conjugation	0032446	8.60E-05

cysteine-type peptidase activity	0008234	8.80E-05
organelle organization	0006996	9.10E-05
microtubule cytoskeleton	0015630	9.90E-05
mitotic cell cycle checkpoint	0007093	1.00E-04
cell cycle checkpoint	0000075	1.00E-04
transcription initiation from RNA polymerase II promoter	0006367	1.00E-04
regulation of cellular biosynthetic process	0031326	1.00E-04
regulation of biosynthetic process	0009889	1.00E-04
DNA-dependent DNA replication	0006261	1.00E-04
pore complex	0046930	1.00E-04
metal ion binding	0046872	1.00E-04
nucleotidyltransferase activity	0016779	1.00E-04
small GTPase regulator activity	0030695	1.00E-04
protein transporter activity	0008565	1.00E-04
DNA-directed DNA polymerase activity	0003887	1.00E-04
Cell cycle	0007049	1.60E-04
T cell receptor signalling pathway	0050852	1.10E-03
MAPKinase Signaling Pathway	0008349	3.60E-03

Supplementary data table 4a GO of gene sets positively enriched in the 30-45 age group

Term	GO Term number	P.Value
multicellular organismal process	0032501	3.00E-15
sequence-specific DNA binding	0043565	2.10E-13
extracellular region	0005576	6.10E-12
transcription factor activity	0000989	3.10E-11
anatomical structure morphogenesis	0009653	2.70E-09
system process	0003008	3.10E-09
anatomical structure development	0048856	3.80E-09
developmental process	0032502	7.30E-09
organ morphogenesis	0009887	1.20E-08
system development	0048731	1.30E-08
transcription regulator activity	0030528	2.10E-08
neuron differentiation	0030182	2.50E-08
multicellular organismal development	0007275	2.80E-08
neurogenesis	0022008	3.40E-08
organ development	0048799	5.80E-08
generation of neurons	0048699	7.00E-08
G-protein coupled receptor protein signaling pathway	0007186	1.10E-07
cell surface receptor linked signal transduction	0007166	1.40E-07
cell development	0048468	2.50E-07
cell fate commitment	0045165	4.40E-07
voltage-gated ion channel activity	0005244	6.90E-07
voltage-gated channel activity	0022832	6.90E-07
embryonic development ending in birth or egg hatching	0009792	7.50E-07
neurological system process	0050877	9.10E-07
cellular developmental process	0048869	1.00E-06
hormone activity	0005179	1.00E-06
cell differentiation	0030154	1.10E-06
substrate specific channel activity	0022838	1.60E-06
ion channel activity	0005216	2.30E-06
regulation of developmental process	0050793	2.50E-06
sensory organ development	0007423	2.60E-06
extracellular region part	0044421	2.70E-06
chordate embryonic development	0043009	2.90E-06
regulation of biological process	0050789	3.20E-06
embryonic skeletal system development	0048706	3.40E-06
regulation of growth	0001558	3.40E-06
axonogenesis	0007409	3.60E-06
gated channel activity	0005244	4.10E-06

positive regulation of biological process	0048518	4.60E-06
nervous system development	0007399	5.50E-06
neurite morphogenesis	0048812	6.60E-06
G-protein coupled receptor activity	0004930	8.60E-06
cell morphogenesis involved in neuron differentiation	0048667	8.70E-06
embryonic skeletal system morphogenesis	0048704	1.20E-05
neuron development	0048666	1.20E-05
embryonic development	0009790	1.20E-05
potassium ion binding	0030955	1.20E-05
cell projection morphogenesis	0048858	1.50E-05
neuropeptide signaling pathway	0007218	1.60E-05
regulation of nervous system development	0051960	1.60E-05
biological regulation	0051960	1.70E-05
growth	0040007	1.90E-05
cell morphogenesis involved in differentiation	0000904	2.00E-05
regulation of transcription from RNA polymerase II promoter	0006357	2.00E-05
neuron fate commitment	0048663	2.40E-05
potassium channel activity	0005267	2.70E-05
neurite development	0031175	2.90E-05
positive regulation of cellular process	0048522	3.00E-05
regulation of cellular process	0050794	3.00E-05
regulation of cellular biosynthetic process	0031326	3.20E-05
cell-cell signaling	0007267	4.40E-05
positive regulation of developmental process	0051094	6.70E-05
regulation of cell development	0060284	7.30E-05
regulation of nucleobase, nucleoside, nucleotide and nucleic acid metabolic process	0019219	7.60E-05
alkali metal ion binding	0031420	7.60E-05
embryonic morphogenesis	0048598	8.50E-05
regulation of neuron differentiation	0045664	9.00E-05
skeletal system morphogenesis	0048705	1.00E-04
cation channel activity	0005261	1.00E-04
neuropeptide receptor activity	0008188	1.00E-04
Chondroitin sulfate biosynthesis	0030206	3.10E-03

Supplementary data table 4b GO of gene sets negatively enriched in the 30-45 age group

NAME	NES	NOM p-val
KEGG_MTOR_SIGNALING_PATHWAY	3.98	0.00
BIOPOLYMER_METABOLIC_PROCESS	3.98	0.00
ORGANELLE_PART	3.48	0.00
INTRACELLULAR_ORGANELLE_PART	3.43	0.00
REACTOME_CELL_CYCLE_MITOTIC	3.26	0.00
NUCLEUS	3.09	0.00
MICROTUBULE_CYTOSKELETON	2.96	0.00
PROTEIN_SERINE_THREONINE_KINASE_ACTIVITY	2.94	0.00
TRANSFERASE_ACTIVITY_TRANSFERRING_PHOSPHORUS_CONTAINING_GROUPS	2.81	0.00
CHR4Q31	2.79	0.00
NUCLEOPLASM	2.72	0.00
NUCLEAR_PART	2.72	0.00
PROTEIN_MODIFICATION_PROCESS	2.60	0.00
BIOPOLYMER_MODIFICATION	2.59	0.00
NUCLEOBASENUCLEOSIDENUCLEOTIDE_AND_NUCLEIC_ACID_METABOLIC_PROCESS	2.58	0.00
MICROTUBULE_ORGANIZING_CENTER	2.56	0.00
CHR1P22	2.56	0.00
REACTOME_LATE_PHASE_OF_HIV_LIFE_CYCLE	2.56	0.00
DNA_METABOLIC_PROCESS	2.51	0.00
RECEPTOR_ACTIVITY	-2.51	0.000
OXIDOREDUCTASE_ACTIVITY_ACTING_ON_CH_OH_GROUP_OF_DONORS	-2.53	0.000
INTEGRAL_TO_PLASMA_MEMBRANE	-2.63	0.000
CHR11Q12	-2.64	0.000
G_PROTEIN_COUPLED_RECEPTOR_ACTIVITY	-2.69	0.000
TGFBETA EARLY UP	-2.70	0.000
INTRINSIC_TO_PLASMA_MEMBRANE	-2.72	0.000
MULTICELLULAR_ORGANISMAL_DEVELOPMENT	-2.78	0.000
REACTOME_RNA_POLYMERASE_I_PROMOTER_OPENING	-2.80	0.000
SYSTEM_PROCESS	-2.81	0.000
SYSTEM_DEVELOPMENT	-2.82	0.000
REACTOME_PACKAGING_OF_TELOMERE_ENDS	-2.83	0.000
ANATOMICAL_STRUCTURE_DEVELOPMENT	-2.85	0.000
CELL_CELL_SIGNALING	-2.86	0.000
PLASMA_MEMBRANE_PART	-2.89	0.000
KEGG_NEUROACTIVE_LIGAND_RECEPTOR_INTERACTION	-2.97	0.000
CHR17Q25	-3.01	0.000
REACTOME_PEPTIDE_LIGAND_BINDING_RECEPTORS	-3.02	0.000
REACTOME_G_ALPHA_I_SIGNALLING_EVENTS	-3.02	0.000
REACTOME_DOWNSTREAM_EVENTS_IN_GPCR_SIGNALING	-3.03	0.000
CHR16P11	-3.04	0.000
CHR1P36	-3.05	0.000

KEGG_SYSTEMIC_LUPUS_ERYTHEMATOSUS	-3.06	0.000
CHR16P13	-3.08	0.000
RHODOPSIN_LIKE_RECEPTOR_ACTIVITY	-3.10	0.000
EXTRACELLULAR_SPACE	-3.12	0.000
G_PROTEIN_COUPLED_RECEPTOR_PROTEIN_SIGNALING_PATHWAY	-3.12	0.000
PLASMA_MEMBRANE	-3.20	0.000
CHR17P13	-3.49	0.000
CHR11Q13	-3.76	0.000
EXTRACELLULAR_REGION_PART	-3.79	0.000
REACTOME_CLASS_A1_RHODOPSIN_LIKE_RECEPTORS	-3.85	0.000
OXIDOREDUCTASE_ACTIVITY	-3.88	0.000
EXTRACELLULAR_REGION	-4.04	0.000
CHR19Q13	-4.16	0.000
REACTOME_GPCR_LIGAND_BINDING	-4.60	0.000
CHR19P13	-4.83	0.000
KEGG_OLFACTORY_TRANSDUCTION	-5.67	0.000
REACTOME_OLFACTORY_SIGNALING_PATHWAY	-5.81	0.000

Supplementary data Table 4c GSEA of gene sets positively and negatively enriched in the 30-45 age group

Cadmium Ion Binding	9.40E-11	200
Antigen Processing And Presentation Of Peptide Via Mhc Class Ii	1.40E-05	36
Immune System Process	3.20E-05	2.8
Copper Ion Binding	3.60E-05	11
Basal Part Of Cell	5.90E-05	23
IgG Binding	6.00E-05	58
Endopeptidase Inhibitor Activity	6.30E-05	6.5
B Cell Mediated Immunity	6.70E-05	9.8
Peptidase Inhibitor Activity	9.20E-05	6.2
Response To Muscle Activity	1.30E-04	Inf
Mhc Class Ii Protein Complex	1.30E-04	42
Myeloid Cell Differentiation	1.50E-04	5.7
Inflammatory Response	2.10E-04	3.7
Response To Wounding	2.20E-04	3.2
Enzyme Inhibitor Activity	2.40E-04	4.3
Acute Inflammatory Response	2.80E-04	7.4
Antigen Processing And Presentation	3.10E-04	9.5
Immune Response	3.20E-04	2.7
Hemidesmosome	3.20E-04	190
Immunoglobulin Receptor Activity	3.20E-04	190
Cell Differentiation	3.70E-04	2.2
Pattern Recognition Receptor Activity	3.70E-04	26
Cellular Developmental Process	4.00E-04	2.2
Regulation Of Cell Differentiation	4.30E-04	3.4
T Cell Selection	4.60E-04	24
Immunoglobulin Binding	4.60E-04	24
Lymphocyte Mediated Immunity	5.20E-04	6.5
Hematopoiesis	5.50E-04	3.8
Immunoglobulin Mediated Immune Response	5.60E-04	8.2
Regulation Of Immune System Process	5.60E-04	3.6
Response To Biotic Stimulus	7.10E-04	3.4
Regulation Of Developmental Process	8.00E-04	2.3
Basal Plasma Membrane	8.00E-04	19
Mhc Protein Binding	8.00E-04	19
Adaptive Immune Response	8.20E-04	5.9
Response To Other Organism	8.70E-04	3.9
Hemopoietic Or Lymphoid Organ Development	9.40E-04	3.6

Supplementary data table 5a GO of transcripts changing across the lifespan using a regression analysis

NAME	NES	NOM p-val
NUCLEAR_PART	4.64	0.000
PROGRAMMED_CELL_DEATH	4.56	0.000
REGULATION_OF_TRANSCRIPTION	4.49	0.000
APOPTOSIS_GO	4.48	0.000
STRUCTURAL_CONSTITUENT_OF_RIBOSOME	4.44	0.000
TRANSCRIPTION_FROM_RNA_POLYMERASE_II_PROMOTER	4.43	0.000
RNA_BINDING	4.37	0.000
ORGANELLE_MEMBRANE	4.36	0.000
REGULATION_OF_PROGRAMMED_CELL_DEATH	4.14	0.000
OXIDOREDUCTASE_ACTIVITY	4.10	0.000
MITOCHONDRION	4.09	0.000
STRUCTURAL_MOLECULE_ACTIVITY	4.07	0.000
REGULATION_OF_APOPTOSIS	4.06	0.000
EXTRACELLULAR_REGION	3.97	0.000
CELLULAR_LOCALIZATION	3.92	0.000
INTRACELLULAR_TRANSPORT	3.92	0.000
REGULATION_OF_RNA_METABOLIC_PROCESS	3.90	0.000
ORGANELLE_LUMEN	3.89	0.000
MEMBRANE_ENCLOSED_LUMEN	3.87	0.000
ESTABLISHMENT_OF_CELLULAR_LOCALIZATION	3.86	0.000
NEGATIVE_REGULATE_CELLULAR_METABOLIC_PROCESS	3.84	0.000
REGULATION_OF_DEVELOPMENTAL_PROCESS	3.83	0.000
EXTRACELLULAR_REGION_PART	3.80	0.000
NEGATIVE_REGULATION_OF_METABOLIC_PROCESS	3.79	0.000
NUCLEAR_LUMEN	3.78	0.000
BIOSYNTHETIC_PROCESS	3.78	0.000
REGULATION_OF_TRANSCRIPTIONDNA_DEPENDENT	3.76	0.000
CELL_PROLIFERATION_GO_0008283	3.74	0.000
IDENTICAL_PROTEIN_BINDING	3.74	0.000
ORGANELLE_ORGANIZATION_AND_BIOGENESIS	3.72	0.000
REGULATION_OF_BIOLOGICAL_QUALITY	3.72	0.000
VESICLE_MEDIATED_TRANSPORT	3.70	0.000
ENDOMEMBRANE_SYSTEM	3.66	0.000
CELLULAR_BIOSYNTHETIC_PROCESS	3.63	0.000
REGULATION_OF_TRANSCRIPTION_FROM_RNA_POLYMERAS E_II_PROMOTER	3.63	0.000
MACROMOLECULAR_COMPLEX_ASSEMBLY	3.62	0.000
IMMUNE_SYSTEM_PROCESS	3.62	0.000
NEGATIVE_REGULATION_OF_TRANSCRIPTION	3.62	0.000
PROTEIN_KINASE_CASCADE	3.60	0.000
NEGATIVE_REGULATION_OF_APOPTOSIS	3.56	0.000
NEGATIVE_REGULATION_OF_PROGRAMMED_CELL_DEATH	3.55	0.000

CELLULAR_COMPONENT_ASSEMBLY	3.54	0.000
CELLULAR_LIPID_METABOLIC_PROCESS	3.54	0.000
REGULATION_OF_SIGNAL_TRANSDUCTION	3.52	0.000
ANTI_APOPTOSIS	3.48	0.000
REGULATION_OF_CELL_PROLIFERATION	3.45	0.000
ENDOPLASMIC_RETICULUM	3.43	0.000
CELL_FRACTION	3.43	0.000
NEGATIVE_REGULATION_OF_NUCLEOBASENUCLEOSIDENUCLEOTIDE_AND_NUCLEIC_ACID_METABOLIC_PROCESS	3.42	0.000
TRANSLATION	3.40	0.000
MACROMOLECULE_LOCALIZATION	3.38	0.000
ENZYME_REGULATOR_ACTIVITY	3.38	0.000
TRANSCRIPTION_FACTOR_BINDING	3.36	0.000
ORGANELLE_ENVELOPE	3.35	0.000
IMMUNE_RESPONSE	3.34	0.000
NUCLEOPLASM	3.34	0.000
PROTEINACEOUS_EXTRACELLULAR_MATRIX	3.31	0.000
EXTRACELLULAR_MATRIX	3.31	0.000
RNA_PROCESSING	3.30	0.000
NEGATIVE_REGULATION_OF_DEVELOPMENTAL_PROCESS	3.26	0.000
MACROMOLECULE_BIOSYNTHETIC_PROCESS	3.23	0.000
LIPID_METABOLIC_PROCESS	3.22	0.000
ENVELOPE	3.21	0.000
ORGANIC_ACID_METABOLIC_PROCESS	3.20	0.000
EXCRETION	3.19	0.000
MEMBRANE_FRACTION	3.14	0.000
RNA_POLYMERASE_II_TRANSCRIPTION_FACTOR_ACTIVITY	3.14	0.000
MULTI_ORGANISM_PROCESS	3.13	0.000
CARBOXYLIC_ACID_METABOLIC_PROCESS	3.13	0.000
I_KAPPAB_KINASE_NF_KAPPAB_CASCADE	3.12	0.000
TRANSCRIPTION_COFACTOR_ACTIVITY	3.12	0.000
CYTOSOL	3.11	0.000
REGULATION_OF_MOLECULAR_FUNCTION	3.10	0.000
CYTOSKELETON	3.07	0.000
PROTEIN_LOCALIZATION	3.06	0.000
RESPONSE_TO_STRESS	3.06	0.000
POST_TRANSLATIONAL_PROTEIN_MODIFICATION	3.06	0.000
PROTEIN_BINDING_BRIDGING	3.05	0.000
POSITIVE_REGULATION_OF_SIGNAL_TRANSDUCTION	3.04	0.000
CYTOSKELETAL_PROTEIN_BINDING	3.01	0.000
GTPASE_REGULATOR_ACTIVITY	3.01	0.000
REGULATION_OF_I_KAPPAB_KINASE_NF_KAPPAB_CASCADE	3.00	0.000

Supplementary data table 5b. Gene Set Enrichment Analysis upregulated with increasing age using a regression analysis
